# Data-Driven Asthma Endotypes Defined from Blood Biomarker and Gene Expression Data

**DOI:** 10.1371/journal.pone.0117445

**Published:** 2015-02-02

**Authors:** Barbara Jane George, David M. Reif, Jane E. Gallagher, ClarLynda R. Williams-DeVane, Brooke L. Heidenfelder, Edward E. Hudgens, Wendell Jones, Lucas Neas, Elaine A. Cohen Hubal, Stephen W. Edwards

**Affiliations:** 1 National Health and Environmental Effects Research Laboratory, U.S. Environmental Protection Agency, Research Triangle Park, North Carolina, United States of America; 2 National Center for Computational Toxicology, U.S. Environmental Protection Agency, Research Triangle Park, North Carolina, United States of America; 3 National Health and Environmental Effects Research Laboratory—Environmental Public Health Division, U.S. Environmental Protection Agency, Research Triangle Park, North Carolina, United States of America; 4 National Health and Environmental Effects Research Laboratory—Integrated Systems Toxicology Division, U.S. Environmental Protection Agency, Research Triangle Park, North Carolina, United States of America; 5 Department of Bioinformatics, Expression Analysis, a Quintiles company, Durham, North Carolina, United States of America; 6 Office of Research and Development, U.S. Environmental Protection Agency, Research Triangle Park, North Carolina, United States of America; Cincinnati Children’s Hospital Medical Center, UNITED STATES

## Abstract

The diagnosis and treatment of childhood asthma is complicated by its mechanistically distinct subtypes (endotypes) driven by genetic susceptibility and modulating environmental factors. Clinical biomarkers and blood gene expression were collected from a stratified, cross-sectional study of asthmatic and non-asthmatic children from Detroit, MI. This study describes four distinct asthma endotypes identified via a purely data-driven method. Our method was specifically designed to integrate blood gene expression and clinical biomarkers in a way that provides new mechanistic insights regarding the different asthma endotypes. For example, we describe metabolic syndrome-induced systemic inflammation as an associated factor in three of the four asthma endotypes. Context provided by the clinical biomarker data was essential in interpreting gene expression patterns and identifying putative endotypes, which emphasizes the importance of integrated approaches when studying complex disease etiologies. These synthesized patterns of gene expression and clinical markers from our research may lead to development of novel serum-based biomarker panels.

## Introduction

More than 20 million Americans have asthma, including approximately 7 million children under the age of 18. The cost of treating asthma in children under 18 in the United States is estimated at $3.2 billion per year [1,2]. Recently, there has been an increased scrutiny of the heterogeneity of clinical disease [3,4] and mechanistically distinct endophenotypes, or “endotypes” [5–7]. Most studies, however, rely heavily on conventional clinical diagnostic criteria and a handful of well-established biomarkers [3,4,6–10]. These approaches are limited because the molecular mechanisms underlying different asthma etiologies are as yet inadequately described and remain an area of active research [2,11–13]. New integrative, systems-based approaches can better define the functional and regulatory pathways that play central roles in respiratory pathophysiology [2].

Several studies have leveraged genomics [11,14,15] or proteomics [16] data to better describe the mechanisms underlying different asthma endotypes. Studies using airway epithelial cells identified potential endotypes of asthma [17], evaluated effects on corticosteroid treatment [18], and identified potential biomarkers [19]. Transcriptional phenotypes from induced sputum samples refined the knowledge of distinct molecular mechanisms associated with different asthma endotypes [14]. Genes [15] and proteins [16] have been previously identified from blood that represent potential biomarkers for asthma.

**Fig 1:**
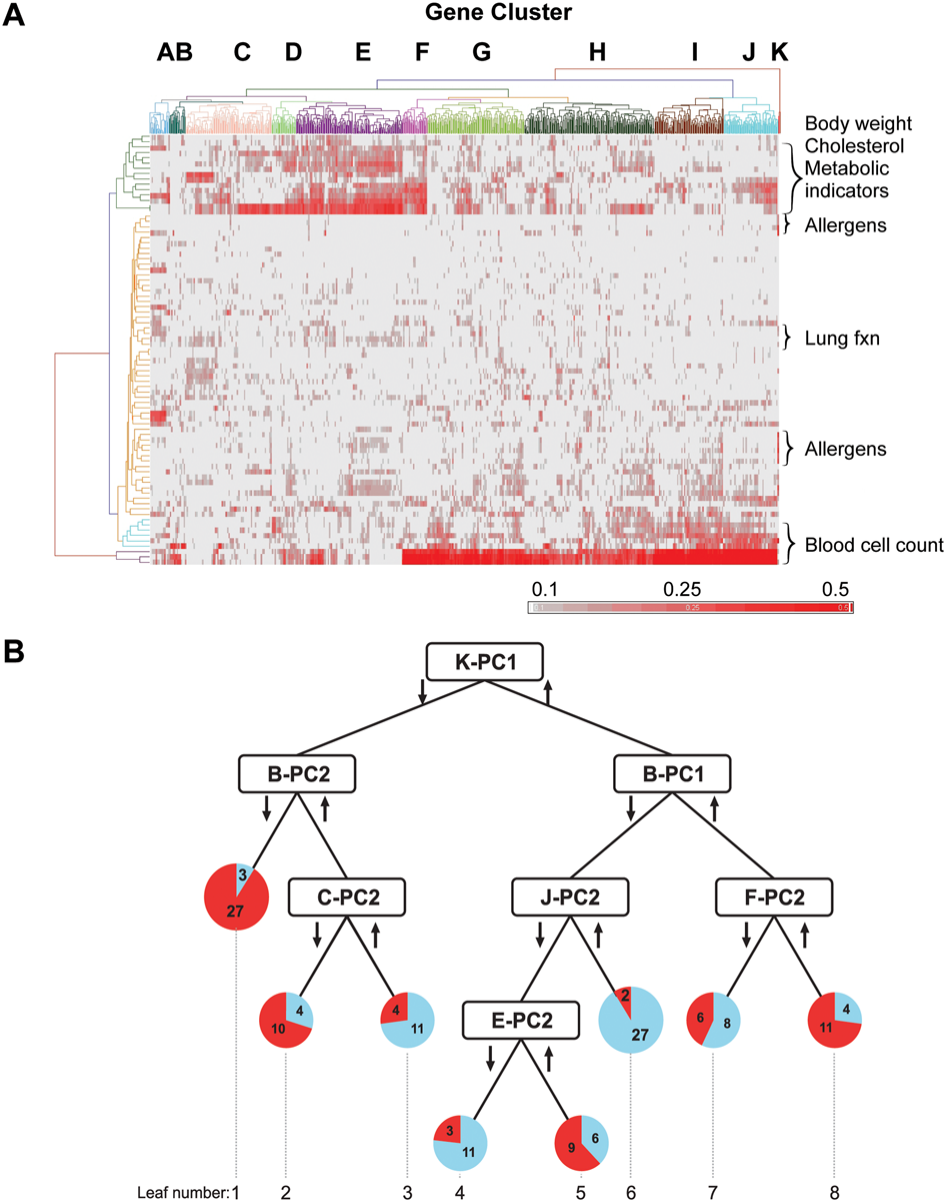
Data integration and reduction to build decision tree. Adapted from [23]. (A) Heat map shows absolute value of the Pearson correlations between 901 genes (X axis) and 81 clinical biomarkers (Y axis) for the 192 study subjects. Hierarchical clustering yielded 11 gene clusters labeled A-K with the corresponding gene lists provided in S5 Table. The clinical biomarkers are listed in S6 Table along with their dendrogram-clustered groupings. (B) Decision tree shows partitioning of the 146 subjects with unambiguous asthma status into mechanistically distinct asthmatic and non-asthmatic leaves based on metagenes developed by dimension reduction of the gene clusters using principal component analysis. The metagenes are labeled by gene cluster and principal component (e.g., K-PC1 represents gene cluster K, principal component 1). Arrows represent whether subjects were above or below the decision tree’s entropy-based cutpoint. Pie charts for each leaf show the number of asthmatics (red) and non-asthmatics (blue). Geometric means of selected clinical biomarkers per leaf are provided in S3 and S4 Tables.

The Mechanistic Indicators of Childhood Asthma (MICA) study collected clinical and blood gene expression biomarkers on a cohort of 192 predominantly African American children from Detroit, MI with and without asthma [20]. Despite a higher prevalence of asthma in low-income and minority children in the U.S., African Americans represent one of the least studied races with regards to asthma [21,22]. Simple clusterings of subjects by either the clinical biomarkers or gene expression alone show no differentiation between asthmatics and non-asthmatics (S1 Fig.). The objective of our study is to differentiate asthmatics from non-asthmatics using a systems-based decision tree approach that incorporates gene expression and clinical biomarker measurements to define potential asthmatic endotypes (Fig. 1).

## Results

Fig. 1A summarizes the Pearson correlations of the gene expression and clinical biomarkers, which yielded 11 gene clusters (A-K) based on shared biomarker correlations. Summarizing the gene expression from each cluster using principal component analysis resulted in 2–5 metagenes per cluster, which were all combined to serve as features for decision tree construction as described in the methods. The result was an optimized tree (Fig. 1B) comprised of 7 metagenes that segregated asthmatics from non-asthmatics with individual leaves representing putative asthma endotypes (Leaves 1, 2, 5, and 8). Building the decision tree with features that aggregated information from clusters of multiple genes based on their correlation with clinical markers maximized the mechanistic information available for interpreting the putative endotypes [23].

**Fig 2:**
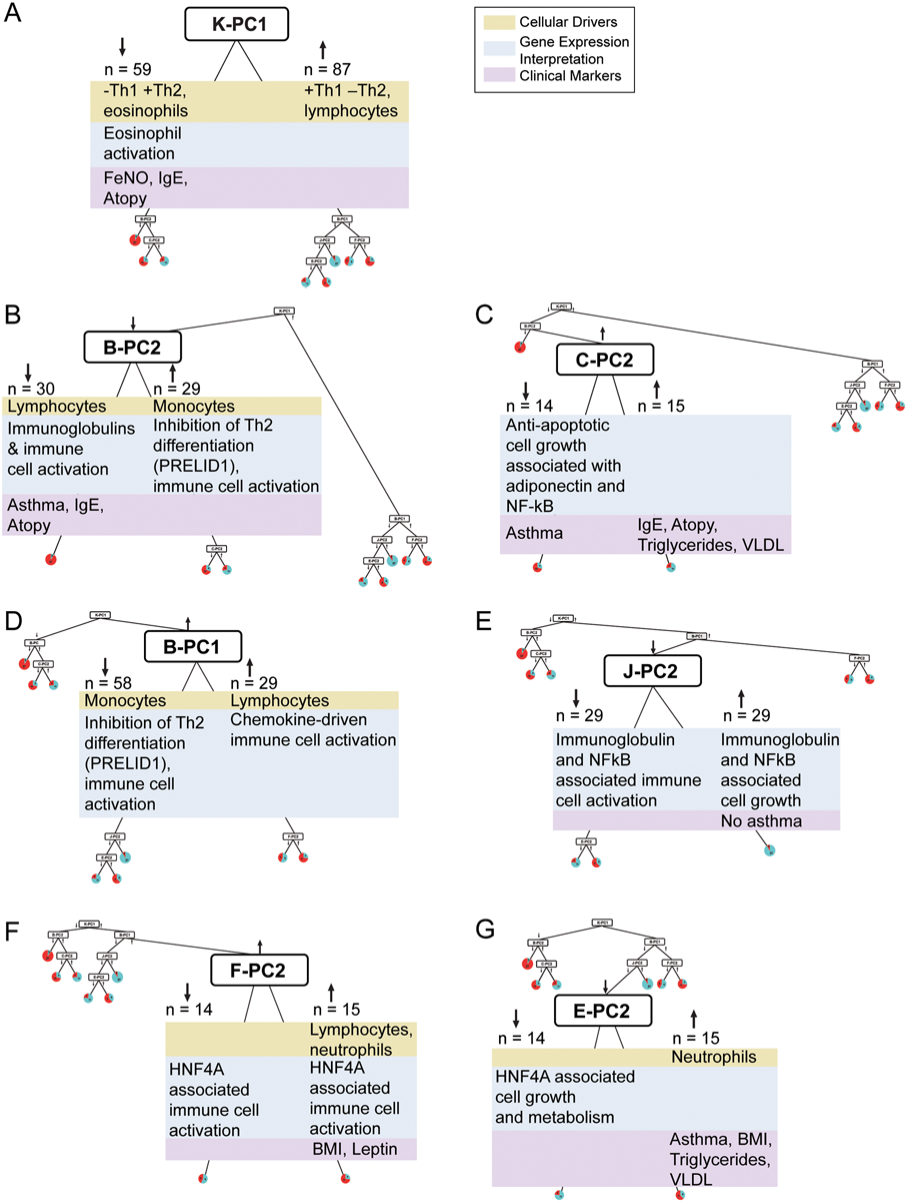
Mechanistic interpretation of the decision tree. Cellular drivers were determined by using linear regression as described in the methods and summarized in S1 Table. The results are summarized in green boxes. Gene expression changes were interpreted using Ingenuity Pathway Analysis (IPA). The top networks from IPA are listed in S2 Table along with their significance scores. All networks that were considered as part of the functional interpretation are included as S2–S9 Figs. The final functional summaries from this analysis are shown in blue boxes. Clinical biomarkers (S3 and S4 Tables) correlated with the key genes from each metagene are shown in the purple boxes; atopy is based on allergen-specific IgE levels (S3 Table, Phadiatop) and IgE represents total serum IgE. (A) K-PC1, no IPA network (B) B-PC2, S3 Fig. (C) C-PC2, S4 Fig. (D) B-PC1, S2 Fig. (E) J-PC2, S8 and S9 Figs. (F) F-PC2, S7 Fig. (G) E-PC2, S5 and S6 Figs.

Since each blood cell type has a distinct gene expression pattern, linear regression analysis was used to account for changes in measured gene expression due to changes in the relative proportions of the cell types. S1 Table shows three metagenes markedly associated with blood cell type (Adjusted R^2^ > 0.1). The biological pathways underlying each metagene were identified via Ingenuity Pathways Analysis (Ingenuity Systems, www.ingenuity.com). S2 Table lists all the networks that were evaluated (S2–S9 Figs.). A number of clinical biomarkers were significantly correlated with key genes underlying each metagene (Fig. 1A). These clinical biomarkers (S3 and S4 Tables) were also considered in the interpretation of the tree. The biomarkers, together with the biological pathways inferred from the gene expression, provided new mechanistic information underpinning the distinct endotypes. Key insights from each data stream are summarized for each of the 7 metagenes (Fig. 2).

### Eosinophilia

The initial branch of the tree is based on the aggregate gene expression summarized in the K-PC1 (cluster K, first principal component) metagene. K-PC1 separated subjects into two distinct groups: those on the left hand side (Leaves 1–3) have a high incidence of eosinophilia (defined as > 0.4 K eosinophils/μL), whereas those on the right hand side (Leaves 4–8) are almost exclusively non-eosinophilic (Fig. 2A and S3 Table). These results suggest that the two putative endotypes on the left hand side (Leaves 1 & 2) would be classified as Th2-high asthmatics whereas the two putative endotypes on the right hand side (Leaves 5 & 8) would be Th2-low asthmatics [6,11,17,24].

As shown in Fig. 2A, all three data streams (cellular drivers, gene expression, clinical markers) help to explain this split. Linear regression found eosinophils and lymphocytes accounted for the majority of this metagene’s variation (Adjusted R^2^ = 0.62) and eosinophils as its primary contributor, with parameter estimate-0.43 (S1 Table). The gene cluster derived from K-PC1 (Fig. 1A) included only three genes: CAT, RNASE2, and CLC, and all three genes have a known role in eosinophil activation. Charcot-Leyden crystal protein (CLC) is a lysophospholipase expressed primarily in eosinophils and basophils and is associated with inflammation in general and eosinophil activation in particular. Polymorphisms in CLC are associated with allergic rhinitis [25]. RNASE2 and catalase (CAT) are highly expressed in eosinophils and are predictive biomarkers of atopy [26] and asthma [27], respectively. All three genes are associated with the left branch of K-PC1 based on the principal component analysis. The cellular and clinical markers associated with Leaves 1–3 (under the left branch for K-PC1) are elevated eosinophil percent and number, increased fractional exhaled nitric oxide (FeNO), and atopy (based on allergen-specific IgE levels), all well-established clinical biomarkers for Th2-mediated immune response. In contrast, Leaves 4–8 (K-PC1, right branch) show no markers related to Th2 influence on asthma status. Following the initial branch, there is no discriminatory power for eosinophils with comparable levels across Leaves 1–3 and Leaves 4–8.

### Eosinophilic Asthmatics Split into Allergic Asthmatics and a Mixed Endotype with Adaptive and Innate Immune Drivers

**Fig 3:**
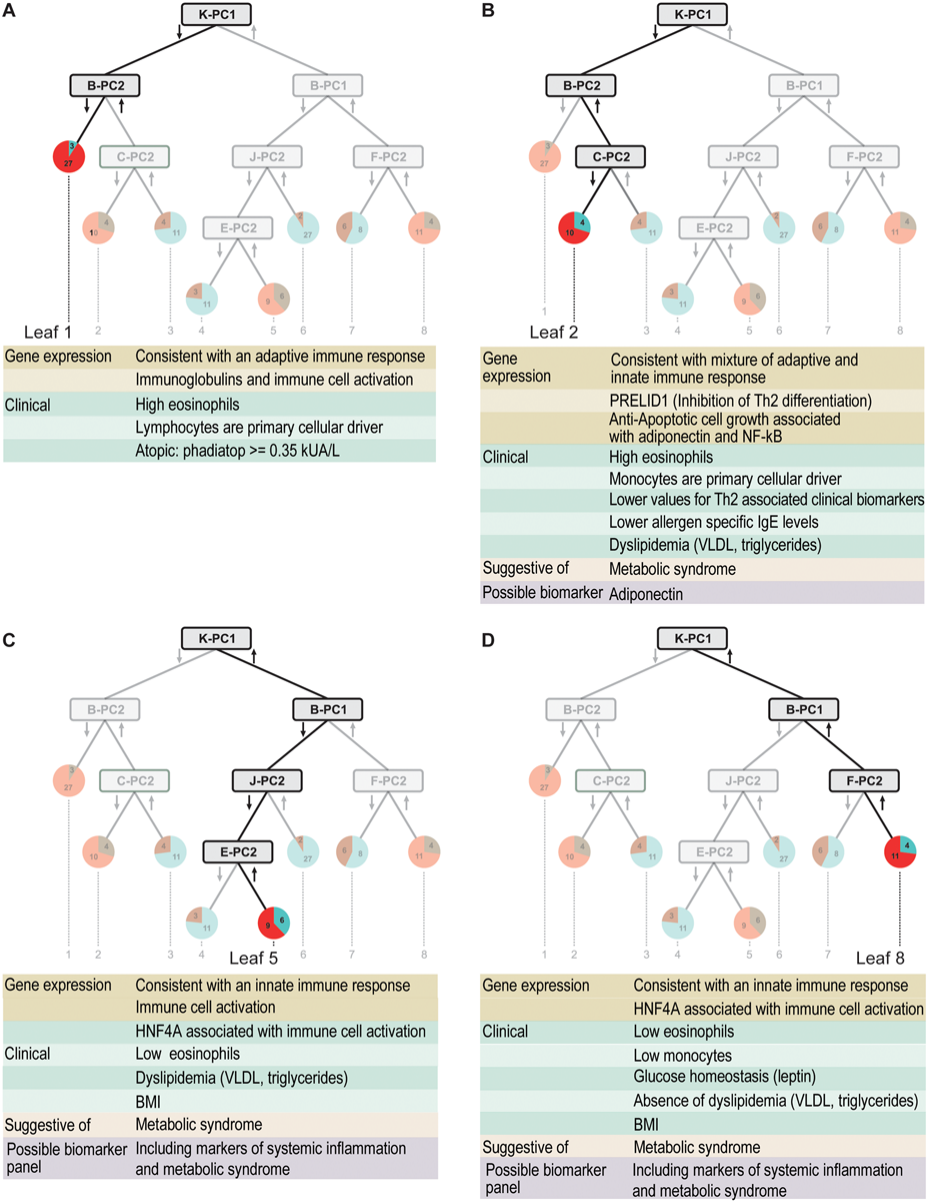
Four distinct asthma endotypes identified by data-driven integration of blood gene expression and clinical biomarkers. Underlying mechanistic information is suggestive of metabolic syndrome and potential biomarkers.

The two eosinophilic asthma endotypes from the left hand side of the tree can be further subdivided into an atopic asthmatic endotype (Fig. 3A) primarily characterized by high eosinophils and established markers for atopy (S3 Table) and a mixed eosinophilic and neutrophilic endotype (Fig. 3B) [11]. The atopic asthmatics in Leaf 1 are defined entirely by metagene B-PC2 (Fig. 2B). The mechanistic interpretation once again matches the clinical characteristics with lymphocytes emerging as the primary cellular driver (S1 Table) and gene expression changes suggestive of an adaptive immune response (S3 Fig.). The Leaf 2 endotype is defined by the combined influence of B-PC2 (Fig. 2B) and C-PC2 (Fig. 2C). In contrast to Leaf 1, the B-PC2 gene expression (S3 Fig.) and cellular driver (monocytes, S1 Table) in this case are more consistent with an innate rather than adaptive immune response. A particularly notable gene is PRELID1, which has previously been shown to inhibit Th2 cell development and may explain the lower values for Th2 associated clinical biomarkers in Leaves 2 and 3 when compared with Leaf 1. The C-PC2 gene annotations (S4 Fig.) and associated clinical biomarkers (very low density lipoproteins (VLDL), triglycerides) suggest an influence of metabolic syndrome in determining this asthmatic endotype. However, the clinical biomarkers are not appreciably different between the two leaves (S4 Table) suggesting the need for new biomarkers. The inclusion of the adiponectin receptor in the C-PC2 gene signature points to adiponectin as a possible biomarker of importance for identifying the Leaf 2 asthma endotype.

B-PC2 (Fig. 2B) separates atopic asthmatics (Fig. 3A) from the other eosinophilic subjects (Leaves 2–3). Lymphocytes and monocytes are the cellular drivers for this metagene, accounting for approximately a third of its variation (Adjusted R^2^ = 0.37), and are similar in magnitude of their parameter estimates (S1 Table). The B-PC2 network (S3 Fig.) has four genes (IGHM, S100A10, EIF4A1, TCL1A) associated with the left branch leading to Leaf 1. While S100A10 and EIF4A1 are involved in more general cellular functions, IGHM and TCL1A are specific to T cell maturation and adaptive immune response. Equally compelling is the association of PRELID1 with the right branch of B-PC2 where inhibition of Th2 cell development [28] potentially reduces the contribution of adaptive immunity to asthma (Fig. 3B); subjects in Leaf 1 where Th2 cell-activated eosinophils and lymphocytes dominate have the complementary absence of any PRELID1-induced protection. The remaining genes associated with the right branch of B-PC2 relate to chemokine signaling and immune cell activation.

C-PC2 (Fig. 2C) separates the subjects from the right branch of B-PC2 into an asthmatic endotype Leaf 2 characterized by lower allergen specific IgE levels and Leaf 3 characterized by high atopy with low asthma prevalence. The network for the C-PC2 metagene (S4 Fig.) contains three informative hub nodes (i.e. highly connected genes within the network) which potentially play a prominent role in the biological processes underlying the observed gene expression changes. The immunoglobulin hub node suggests underlying adaptive immune responses for the Leaf 2 asthma endotype. A second hub is an NFkB complex, broadly associated with enhanced inflammation associated with adaptive and innate immune response. A third hub underscores the importance of insulin with connections to immunoglobulin, NFkB, and key genes from C-PC2. One of the key genes from the C-PC2 metagene is the adiponectin receptor, which plays a role in glucose homeostasis [29] and is associated with asthma risk in women [30,31]. Two clinical markers associated with metabolic syndrome (triglycerides, VLDL) were significantly correlated with the genes in the C-PC2 network (S4 Fig.); however, there was no substantive difference in the geometric means for these markers between Leaves 2 and 3. The clinical measures associated with atopy (total IgE, allergen-specific IgE) are higher in Leaf 3.

### Non-Eosinophilic Asthma Endotypes, Innate Immunity, and Metabolic Syndrome

The two asthma endotypes (Fig. 3 C, D) falling on the right hand side of the tree show little evidence for involvement of eosinophils or adaptive immunity, which is consistent with a Th2-low classification [11]. Both the B-PC1 (Fig. 2D) and J-PC2 (Fig. 2E) metagene networks contain genes associated with innate immune cell activation (S2 and S8 Figs.); the F-PC2 (Fig. 2F) and E-PC2 (Fig. 2G) metagene networks contain genes associated with metabolic syndrome (S5 and S7 Figs.). In particular, both the F-PC2 and E-PC2 networks contain the HNF4A or MODY1 gene, which has long been associated with Type 2 diabetes [32] but not previously associated with asthma. As with the Leaf 2 endotype on the left hand side of the tree, clinical biomarkers associated with metabolic syndrome (body mass index, leptin, triglycerides, VLDL) are associated with the E-PC2 and F-PC2 metagenes but alone are insufficient to define either asthma endotype. The mechanistic differences between the Leaf 5 (Fig. 3C) and Leaf 8 (Fig. 3D) endotypes appear to be subtle. One interesting clue is the stronger association of Leaf 5 with clinical markers of dyslipidemia (VLDL, triglyderides) and Leaf 8 with glucose homeostasis (leptin). Another clue is the strong influence of monocytes on the B-PC1 metagene corresponding to a noticeable difference in monocyte count in Leaves 4–6 compared with Leaves 7–8.

B-PC1 (Fig. 2D) separates 58 subjects, where approximately one fourth are asthmatic, from 29 subjects, where over half the subjects have asthma. As with its counterpart on the left side of the tree (B-PC2, Fig. 2B), B-PC1 is partially driven (Adjusted R^2^ = 0.35) by monocytes and lymphocytes as indicated by the linear regression results (S1 Table). The two most highly connected hubs in the B-PC-1 network (S2 Fig.) are interferon-gamma and beta-estradiol. The interferon-gamma protein is a potent activator of macrophages, which coupled with the fact that the majority of key genes for this metagene are associated with its left branch, suggests that macrophage activation maybe an important driver for the Leaf 5 asthma endotype (Fig. 3C). This interpretation is further supported by the association of monocytes (a macrophage precursor) with B-PC1’s left branch and consequent higher levels of monocytes in all its corresponding Leaves (4–6) relative to B-PC1’s right branch (Leaves 7 and 8) (S3 Table). Four genes (S100A10, CX3CR1, EIF4A1, PRELID) from the B-PC1 network (S2 Fig.) are associated with regulation of immune cell activation and differentiation. Two other genes (LGALS1, PTP4A2) are generally associated with increased cellular proliferation and migration, both of which play a role in neutrophil and monocyte activation and invasion of these cells to target tissues. Protein complexes including two other key genes from this network (S100A10, ANXA2) also influence macrophage activation by plasmin [33,34].

J-PC2 (Fig. 2E) is the next metagene below B-PC1’s left branch and partitions the corresponding non-asthmatics mostly into Leaf 6, rendering the leaf with the smallest percentage of asthmatics (<10%). The J-PC2 network (S8 Fig.) shares two hub genes (immunoglobulin, NFkB) with C-PC2 but has no genes associated with metabolic syndrome. Instead, the majority of the key genes from this metagene are associated with immune cell activation and cell growth as seen with B-PC1 (e.g. TNFRSF1A, colony stimulating factor 2 receptor (CSF2RB), formyl peptide receptor 1 (FPR1), and several chemokine and cytokine receptors). This network includes integrin (ITGAX), which has been specifically shown to mediate the adherence of monocytes and neutrophils to stimulated endothelial cells, increasing the confidence in the B-PC1 suggestion that a heightened activation state of these cells may increase asthma susceptibility by making them more prone to invasion of the lung tissue. The heightened state of activation is further indicated by the presence of a regulator of cytokine production (NAMPT) and a gene (HCK) previously implicated in respiratory burst, migration, and degranulation in neutrophils.

F-PC2 (Fig. 2F) is the next metagene below B-PC1’s right branch and it partitions subjects into Leaf 7, a mixed group with slightly less than 50% asthmatics, and Leaf 8, a putative asthma endotype. The central hub node in the networks for both E-PC2 (S5 Fig.) and F-PC2 (S7 Fig.) is HNF4A (often referred to as MODY1). Mutations in this gene are associated with Type 2 diabetes [32]. The clinical biomarkers BMI and leptin are correlated with this metagene and are commonly associated with metabolic syndrome, but as for C-PC2, there was no substantive differentiation of subjects into Leaves 7 and 8. F-PC2 is unique among the metagenes associated with metabolic syndrome (the others being C-PC2 & E-PC2) in that the associated biomarker is involved with glucose regulation (leptin) rather than a marker for dyslipidemia (triglycerides, VLDL). The F-PC2 network (S7 Fig.) includes several genes that support the overall theme of a heightened activation state for the innate immune system. A membrane bound cytokine (CKLF) is a potent chemoattractant for neutrophils, monocytes, and lymphocytes; a pair of the key genes (S100A12, S100A9) bind the cytoskeleton and regulate oxidative metabolism in neutrophils [35]. Asthmatic subjects are not partitioned cleanly into Leaves 7 and 8; thus, these two leaves may represent a single asthma endotype. Leaf 7 does have a slightly different profile of clinical markers with the highest total and antigen-specific IgE of all the leaves on the right hand side of the tree. This suggests that Leaf 7 could have something in common with Leaf 2 as well. Despite the ambiguity with this particular leaf, the F-PC2 metagene still provides additional support for the role of both metabolic syndrome and inflammatory cell activation in determining the mechanistic basis underlying an asthma endotype.

E-PC2 (Fig. 2G) defines the one potential asthma endotype (Leaf 5) that falls under the left branch of B-PC1. The central hub node in networks for both E-PC2 (S5 Fig.) and F-PC2 (S7 Fig.) is HNF4A (aka MODY1), which has long been associated with Type 2 diabetes [32]. The key genes for this metagene do not provide the same level of mechanistic information seen with the other metagenes. The majority of the key genes correspond to ribosomal proteins, and all the genes in this network perform general cellular functions relating to cellular proliferation, differentiation, and energetics. E-PC2 shares clinical biomarkers with F-PC2 (BMI) and C-PC2 (triglycerides, VLDL) related to metabolic syndrome.

### Impact of Medication Use

A survey of asthma medication use among the eight leaves resulting from the decision tree analysis (S4 Table) showed no correspondence between the frequency of medication use and any of the resulting endotypes. A similar analysis focusing on different classes of medication (i.e. corticosteroids, leukotriene inhibitors, beta-adrenergic agonists) also showed no relationship between drug class and the putative endotypes. In addition, the close correspondence between the putative endotypes in Leaves 1 and 2 and previous clinical classifications suggest that while medication use may relieve the overt symptoms, the mechanistic biomarkers (e.g. eosinophils, FeNO, total IgE, allergen-specific IgE) are still altered. While medication use does not appear to explain our results, it represents a confounding factor in this analysis. It is interesting to note that all asthmatics falling into leaves with predominantly non-asthmatic subjects (Leaves 3, 4, 6, and 7) were on medications, which could indicate a slight dampening of the molecular markers by the asthma medications. The incidence of daily medication use also tended to be higher for asthmatics in the non-asthmatic leaves. An open question is the impact of medication use on the lack of distinction seen between asthma and no asthma in Leaf 7.

## Discussion

Our novel, multi-step, systems-based decision tree approach using principal components-summarized gene expression and clinical biomarker correlations [23] differentiated asthmatics from non-asthmatics revealing biological pathways that potentially underlie the varied asthmatic endotypes (Fig. 1). The results of this study are for children aged 9 to 13 years and cannot be extrapolated to all ages. In adults, for example, there are higher prevalence rates of other asthma phenotypes and endotypes, such as non-allergic asthma and aspirin-exacerbated asthma [36]. Characteristics of the data-driven derived endotypes from this study are consistent with previously published endotypes based solely on clinical diagnostic criteria [3,4,6,7,9], but our data-driven method provides mechanistic understanding that is not possible when using established clinical markers alone. One theme that emerges from this analysis is the interplay between innate and adaptive immune responses. We clearly see a dominant role for adaptive immunity in Leaf 1, innate immunity in Leaves 5 and 8, with a mixed contribution in Leaf 2 (Fig. 3). Our results also suggest a role for broad systemic inflammation in addition to the localized hyperreactivity in the lung as a major driver for asthma. This was particularly prominent with the innate immune mediators. The role for monocytes in mediating asthma has not been explored to the same degree as neutrophils probably due to the prevalence of resident macrophages in the lung. Our results from blood suggest a prominent role for an enhanced activation state of these circulating cells in at least one of our asthma endotypes.

Our findings are consistent with studies demonstrating that weight loss improves asthma symptoms without significant changes in markers of airway inflammation [37]. Of note, BMI alone is not a predictor of asthma in our study (S4 Table) in contrast with other recent studies [38]; this may be because we are looking at asthma prevalence in children rather than correlates of asthma onset. Our study, among others [39–42], putatively identifies underlying mechanisms linking obesity and asthma through systemic inflammation related to metabolic syndrome and increases the relevance and understanding of clinical findings. This knowledge, coupled with genetic associations of obesity with asthma [43] and targeted mechanistic studies [44], should foster better treatment and diagnosis of these endotypes. An example is the C-PC2 metagene support for adiponectin as an asthma biomarker in addition to its recognized role in metabolic syndrome and chronic obstructive pulmonary disease [45,46]. In addition, the prominence of the HNF-4A gene in the networks for E-PC2 and F-PC2 suggest that polymorphisms in this gene could influence asthma in addition to their known role in diabetes susceptibility.

In addition to providing mechanistic information important for developing new biomarkers, our results provide additional context for interpreting several existing asthma biomarkers. RNASE2 and catalase (CAT) are highly expressed in eosinophils and have been shown to be predictive biomarkers for atopy [26] and asthma [27], respectively. Our data-driven study supports the ATS clinical recommendations regarding the use of fractional exhaled nitric oxide (FeNO) for asthma diagnosis [47,48]. For children, the predictive ability of FeNO is considerably stronger for atopy in allergic non-asthmatics [49], and there is some question regarding clinical significance in adults [50]. Since all three biomarkers are critical players for the top left branch in our tree, our results suggest that they better reflect eosinophilia rather than IgE-mediated atopy or asthma specifically. In our study, FeNO shows no relationship with asthma (S1 Table) when considering either the left or right sides of the tree separately (i.e., after the K-PC1 split on eosinophilia).

Induced sputum eosinophilia has also been used as a biomarker in clinical trials and has proven informative for regulating corticosteroid dose for asthma control [51]. More recently, an assay based on three IL-13 regulated genes showed promise in distinguishing Th2 driven asthma (Th2-high) from alternate mechanisms (Th2-low) [17]. Molecular indicators from airway samples for Th2-low asthmatics have remained elusive. Our results indicate the possibility that airway hyperresponsiveness in these endotypes is elicited by triggers due to a heightened state of alert for circulating innate immune cells. Detection of increased airway inflammation will consequently be restricted to periods of active airway constriction during an asthma attack, highlighting the importance of systemic biomarkers for asthma diagnosis. Given that inhaled corticosteroids are most effective in Th2-high individuals [24,47], our putative endotypes from the right hand side of the decision tree (Leaves 5, 8, and possibly 7) provide important information for development of new therapies and diagnostic biomarkers for this ever-growing population [52,53]. Specifically, our results suggest that a biomarker panel including markers of systemic inflammation as well as metabolic syndrome is needed for better diagnosis of distinct asthma endotypes.

The strong association between our asthma endotypes and both systemic inflammation and metabolic syndrome-associated clinical indicators suggests that asthma incidence for the Th2-low endotypes described here (Leaves 5 and 8) may continue to rise with the worldwide escalation in obesity. Given that inhaled corticosteroids are most effective in Th2-high individuals [24,47], our putative Th2-low endotypes [17,24] add important mechanistic information for development of new therapies and diagnostic biomarkers for this ever-growing population. These proposed endotypes, along with their associations with key biological pathways, should also provide valuable insights for interpreting the continually expanding list of genes putatively identified as genetic risk factors for asthma. Finally, a better understanding of the various asthma endotypes from this and complementary studies provides a scientifically defensible foundation for the evaluation of the many environmental factors influencing each mechanistically distinct endotype. These synthesized patterns of gene expression and clinical markers from our research may lead to development of novel serum-based biomarker panels that have improved sensitivity and specificity in clinical diagnosis of asthma over biomarkers currently available and reflected in conventional studies of asthmatics.

## Materials and Methods

### Study Design/Details of Cohort

Details of the Mechanistic Indicators of Childhood Asthma (MICA) study have been previously published [20]. MICA was a cross-sectional study of a cohort of 205 children comprising two strata: children with asthma and children without asthma selected in an approximately 1:1 ratio. The rationale for including both asthmatics and non-asthmatics was to provide a basis for classifying the individuals, rather than simply clustering asthmatics, in evaluation of several methods for differentiating asthmatics from non-asthmatics [23]. Children aged 9 to 13 years residing in the communities of Detroit, Dearborn, Highland Park, or Hamtramck and who are served by the Henry Ford Health System were eligible for selection into the MICA study. Inclusion criteria for asthmatic children: a parent reported doctor’s diagnosis of asthma, both genders, and all racial/ethnic groups were eligible. Exclusion criteria: medical history or underlying health problems that preclude participation in the protocol per the physician (includes cystic fibrosis, viral bronchiolitis, bronchopulmonary dysplasia, heart disease, vocal cord dysfunction, laryngotracheomalacia, tracheal stenosis, bronchostenosis, or who received oxygen for more than two weeks after birth or at home), history of respiratory illness in the last two weeks, had ever smoked five or more cigarettes, or who had been a carrier of a communicable disease. Study participants completed two health questionnaires and underwent a clinical exam including lung function and analysis of exhaled breath. In addition, blood was drawn from each participant for analysis of clinical biomarkers and gene expression analysis from whole blood. The study design and protocols were approved by the Institutional Review Boards (IRB) at Henry Ford Health System (Detroit, MI), Westat Inc. (Rockville, MD), and the University of North Carolina at Chapel Hill (US EPA’s IRB of record; Chapel Hill, NC). Written consent was obtained from guardians, and written assent was obtained from each child, with an oral review of both consent and assent prior to study enrollment.

Of the 205 participants in the original study, data from 192 were used in the clustering of gene expression and clinical biomarkers (independently of clinical asthma status). Ten of the 205 subjects were excluded because there was insufficient RNA for the gene expression study. Two other subjects were excluded from the analysis because data were mislabeled (one “male” and one “female”) and appeared erroneously in two clear sex-specific clusters in a principal component that explained 62% of the variation in Y-chromosome gene expression. For the remaining subject excluded from the analysis, data were missing for 52 of the 81 clinical biomarkers. Subjects were classified as asthmatic or non-asthmatic based on both clinical records and a parental questionnaire. A child was considered asthmatic if the clinical record showed one or more asthma-related emergency department visits, two or more asthma-related outpatient visits, or two or more asthma-related medications. From the parental questionnaire, a child was considered asthmatic with a parental report of a physician’s diagnosis of asthma. The decision tree was built using data from 146 children (72 asthmatics, 74 non-asthmatics) with concordant parental and clinical information, thus excluding children with conflicting or incomplete asthma status data.

### Collecting clinical biomarker data

See [23] for a detailed description of the clinical biomarker data. The biomarker data include a number of clinical measures of hematologic, immunologic, and cardiopulmonary variables, body size measures, allergen exposure indicators, and characterization (titers and types) of circulating white blood cells. Although individual slices of this rich dataset deserve focused study, the present analysis used data for the 81 biomarkers appropriate for our biomarker-genomic analysis. The biomarkers were chosen for completeness (i.e. missing data minimized), sampling distribution (normality was checked before correlations were calculated), and comparison of our data with expected values from previous studies [23].

### Gene expression analysis

Total RNA from blood collected during observational clinic visits [20] was used for Affymetrix gene expression analysis as previously described [23]. Briefly, blood collected in PAXgene tubes was used for total RNA isolation using DNAase treatment. Blood gene expression was measured by Expression Analysis, Inc. (www.expressionanalysis.com, Durham, NC) using the Affymetrix GeneChip Human Genome U133 Plus 2.0 Array. The raw microarray data were subjected to the Reduction of Invariant Probes (REDI) algorithm (http://www.expressionanalysis.com/images/uploads/tech_notes/REDI_Tech_Note1.pdf) to remove data from unresponsive probes, were MAS5 normalized, and were adjusted for sex because this biomarker dominated the changes in expression seen among the subjects. Finally, genes were filtered using the interquartile range to keep genes whose expression varied across the study population and log2 transformed to yield a roughly Gaussian distribution. Of the more than 56,000 probe sets collected for each subject, a subset of 1,279 was selected for the multi-step decision tree method. Of these, 901 probe sets showed a significant correlation (p < 0.0006) with at least one of the clinical biomarkers; these 901 probe sets were used in this study.

The microarray data from this publication have been submitted to the Gene Expression Omnibus (GEO) repository (http://www.ncbi.nlm.nih.gov/geo/) with identifier GSE35571.

### Data analysis strategy (contextual approach)

Following a detailed evaluation of methods based on the ability to segregate asthmatics from non-asthmatics and provide information regarding the mechanistic drivers underlying the segregation, a novel multi-step process (Fig. 1) was chosen [23]. Briefly, the first step was to calculate the correlation of each clinical biomarker with each gene expression variable for the 192 subjects retained in the study, giving a distribution of Pearson correlation coefficients relating the 81 biomarkers to each of the 901 genes passing the non-specific IQR filter. Second, to reveal patterns in the gene-biomarker correlations, we performed unsupervised heat map clustering (complete linkage; Euclidean distance) using the absolute correlation values [0,1] for all significant biomarker-associated genes (Fig. 1A). Certain biomarkers heavily influenced the heat map clustering of the genes due to the large number of associated genes. These biomarkers included measures of white blood cell types (e.g. percent lymphocytes), nutrition (e.g. triglycerides), body size (e.g. BMI), blood chemistry (e.g. total protein), and immune markers (e.g. interleukin 4). Third, the genes in the 11 groups/clusters (labeled Gene Cluster A-K in Fig. 1A) were used as the basis for constructing metagenes that summarized information content across all gene expression variables in each of the cluster groups. The metagenes were created as 11 linear combinations of the 901 genes, a substantial reduction of the dimensionality of the data, by performing separate principal component analyses (PCA) on the expression data for each gene cluster identified above. This use of PCA was for data reduction [54] and did not factor into the separation of the subjects performed in the fourth step. Metagenes were named according to gene-biomarker cluster and principal component number (e.g. F-PC2 for the second principal component (PC) from the PCA on genes in cluster F). Fourth, in order to identify asthma endotypes, these metagenes were input to a recursive partitioning/decision tree method; the rendered tree was comprised of 7 metagenes selected for optimally partitioning subjects according to biomarker-associated gene expression patterns (Fig. 1B). Finally, interpretation of our decision tree involved 3 main aspects of the MICA data: internal annotation via biomarker-genomic correlation patterns, factor loadings from PCA, and pathway analysis via external annotation (Ingenuity Pathway Analysis).

### Regression analysis of metagenes on cell type

All metagenes identified in the development of the decision tree were evaluated by linear regression to estimate variation in the summarized gene expression attributable to the cell types (eosinophils, lymphocytes, neutrophils, monocytes, basophils). Adjusted R^2^ values for the final models are shown in S1 Table, and these may be mildly inflated as a consequence of partial collinearity among cell type percentage variables. Collinearity diagnostics were performed on the final models [55], and no evidence was found of collinearity with substantive consequence.

Specifically, linear models were fit for metagene expression using stepwise selection criteria of 0.2 for entry and 0.05 to stay in the SAS REG procedure [55]. Collinearity diagnostics were performed on the final models in SAS REG, which follows Belsley, Kuh, and Welsch [56] in its approach and includes calculation of condition indices. Belsley, Kuh, and Welsch suggest that when the condition index is around 10 that weak dependencies may start to affect the regression estimates and when larger than 100 may reflect a fair amount of numerical error. Clusters B-PC2 and B-PC1 each have a condition index of 10.64 paired with very small p-values, so any corresponding variance inflation probably would not change the significance reflected by p-values. F-PC2 has a condition index of 55.62 but an Adjusted R^2^ of only 0.05, so the variation in F-PC2 is only weakly correlated with any of the cell types. An additional model was fit for F-PC2 removing the neutrophils variable; the resulting Adjusted R^2^ was lower (0.0157), lymphocytes were significant (p-value = 0.0463), and the corresponding condition index was 8.24.

### Pathways analysis with Ingenuity Pathways Analysis

Because the metagenes in our decision tree contained summary information from a large set of genes, those genes identified as key (see below) for each metagene were used to help define biological pathways that potentially contribute to mechanistic underpinnings of the different endotypes. Gene lists for each metagene were uploaded to Ingenuity Pathways Analysis (Ingenuity Systems, www.ingenuity.com) to identify these underlying biological pathways. The highest scoring network for each principal component was interpreted for all metagenes, and the second highest network was considered when the difference in score between it and the top network was relatively small (S2 Table). S2–S9 Figs. show all networks evaluated.

Key genes were derived for each metagene from the tree by restricting to genes from the cluster where the absolute value of the loading (a measure of the influence for that gene on the principal component) was greater than 0.1. This value was empirically set to generate lists of the appropriate size (13–52) for downstream pathway analysis without considering gene identity or function. The K-PC1 metagene was excluded from this analysis since there were only three genes in that cluster. Each gene identifier was mapped to its corresponding gene object in the Ingenuity Pathways Knowledge Base. These genes were overlaid onto a global molecular network developed from information contained in the Ingenuity Pathways Knowledge Base. Networks of these focus genes were then algorithmically generated based on their connectivity. The loading of the gene relative to the metagene was used in place of a raw expression score. As a result, genes with a negative loading (green nodes) are positively associated with the left branch (lower values for the metagene) in the decision tree. Genes with a positive loading (red nodes) are associated with the right branch (higher values for the metagene) in the tree. The Functional Analysis of the top scoring network identified the biological functions and/or diseases that were most significant to the genes in the network. The network genes associated with biological functions and/or diseases in the Ingenuity Pathways Knowledge Base were considered for the analysis. Fisher’s exact test was used to calculate a p-value determining the probability that each biological function and/or disease assigned to that network is due to chance alone.

## Supporting Information

S1 Fig Representation of raw data. (A) Heatmap showing the 81 clinical biomarkers (X axis) for all subjects (Y axis). (B) Heatmap showing the gene expression (X axis) for 901 genes used in downstream analyses for all 192 subjects (Y axis). For both panels, values are scaled by column using Z scores. Asthma status based on doctor diagnosis is indicated on the left hand side of the heatmap (red = no asthma, green = asthma, blue = unknown). The total number of asthmatics is 96 and the total number of non-asthmatics is 88. Eight individuals did not have a reported asthma status from the doctor diagnosis.
(PNG)

S2 Fig B-PC1, Network 1. The networks were generated through the use of Ingenuity Pathways Analysis (Ingenuity Systems, www.ingenuity.com). The loading value for each gene was imported in place of an expression value, so green nodes (negative loadings) indicate a gene associated with the left branch (Down) of the metagene whereas red nodes (positive loadings) indicate genes associated with the right branch (Up).
(JPG)

S3 Fig B-PC2, Network 1. The networks were generated through the use of Ingenuity Pathways Analysis (Ingenuity Systems, www.ingenuity.com). The loading value for each gene was imported in place of an expression value, so green nodes (negative loadings) indicate a gene associated with the left branch (Down) of the metagene whereas red nodes (positive loadings) indicate genes associated with the right branch (Up).
(JPG)

S4 Fig C-PC2, Network 1. The networks were generated through the use of Ingenuity Pathways Analysis (Ingenuity Systems, www.ingenuity.com). The loading value for each gene was imported in place of an expression value, so green nodes (negative loadings) indicate a gene associated with the left branch (Down) of the metagene whereas red nodes (positive loadings) indicate genes associated with the right branch (Up).
(JPG)

S5 Fig E-PC2, Network 1. The networks were generated through the use of Ingenuity Pathways Analysis (Ingenuity Systems, www.ingenuity.com). The loading value for each gene was imported in place of an expression value, so green nodes (negative loadings) indicate a gene associated with the left branch (Down) of the metagene whereas red nodes (positive loadings) indicate genes associated with the right branch (Up). See S6 Fig. for the second highest scoring IPA network for E:PC2 since its score was still reasonably high relative to the top scoring network (S3 Table).
(JPG)

S6 Fig E-PC2, Network 2. The networks were generated through the use of Ingenuity Pathways Analysis (Ingenuity Systems, www.ingenuity.com). The loading value for each gene was imported in place of an expression value, so green nodes (negative loadings) indicate a gene associated with the left branch (Down) of the metagene whereas red nodes (positive loadings) indicate genes associated with the right branch (Up). Second highest scoring IPA network for E:PC2 since its score was still reasonably high relative to the top scoring network (S3 Table).
(JPG)

S7 Fig F-PC2, Network 1. The networks were generated through the use of Ingenuity Pathways Analysis (Ingenuity Systems, www.ingenuity.com). The loading value for each gene was imported in place of an expression value, so green nodes (negative loadings) indicate a gene associated with the left branch (Down) of the metagene whereas red nodes (positive loadings) indicate genes associated with the right branch (Up).
(JPG)

S8 Fig J-PC2, Network 1. The networks were generated through the use of Ingenuity Pathways Analysis (Ingenuity Systems, www.ingenuity.com). The loading value for each gene was imported in place of an expression value, so green nodes (negative loadings) indicate a gene associated with the left branch (Down) of the metagene whereas red nodes (positive loadings) indicate genes associated with the right branch (Up). See S9 Fig. for the second highest scoring IPA network for J:PC2 since its score was still reasonably high relative to the top scoring network (S3 Table).
(JPG)

S9 Fig J-PC2, Network 2. The networks were generated through the use of Ingenuity Pathways Analysis (Ingenuity Systems, www.ingenuity.com). The loading value for each gene was imported in place of an expression value, so green nodes (negative loadings) indicate a gene associated with the left branch (Down) of the metagene whereas red nodes (positive loadings) indicate genes associated with the right branch (Up). Second highest scoring IPA network for J:PC2 since its score was still reasonably high relative to the top scoring network (S3 Table).
(JPG)

S1 Table Contribution of cell type to metagene summarized expression. Results from a forward-reverse multi-step regression of each metagene from the asthma decision tree on the white blood cell counts and percentage of each individual cell type. Overall contribution of relative changes in cell type was based on the Adjusted R^2^ coefficient of determination. The sign of the parameter estimate was used to determine the branch of the tree associated with the cell type.
(DOCX)

S2 Table Top scoring Ingenuity Pathways Analysis networks with associated annotations. B-PC1 and F-PC2 had only one significant network each. For all other metagenes, the highest scoring network that was not considered is included and the score shown. Full page views of all networks considered in the interpretation phase are shown in S2-S9 Figs.
(DOCX)

S3 Table Inflammation/allergy-related characteristics of subjects by leaf (*endotype). Geometric means and CIs were calculated using SAS-callable SUDAAN [57]; other statistics were calculated using SAS [58].
(DOCX)

S4 Table Metabolic syndrome-related clinical markers along with lung function and medication use by leaf (*endotype). (DOCX)

S5 Table Gene clusters from Fig. 1A. Gene symbols as of the date of analysis along with Affymetrix probe ids are provided. Clusters are identified by color (from the dendrogram) and letter (from labels) in reference to Fig. 1A of the main manuscript.
(XLSX)

S6 Table Biomarker listing with rows and groups as in Fig. 1A dendogram. (DOCX)
