# Supplementary figures and images for: Data-Driven Asthma Endotypes Defined from Blood Biomarker and Gene Expression Data
Source: PLoS One. 2015 Feb 2;10(2):e0117445. doi: 10.1371/journal.pone.0117445 (PMC4314082; doi:10.1371/journal.pone.0117445)

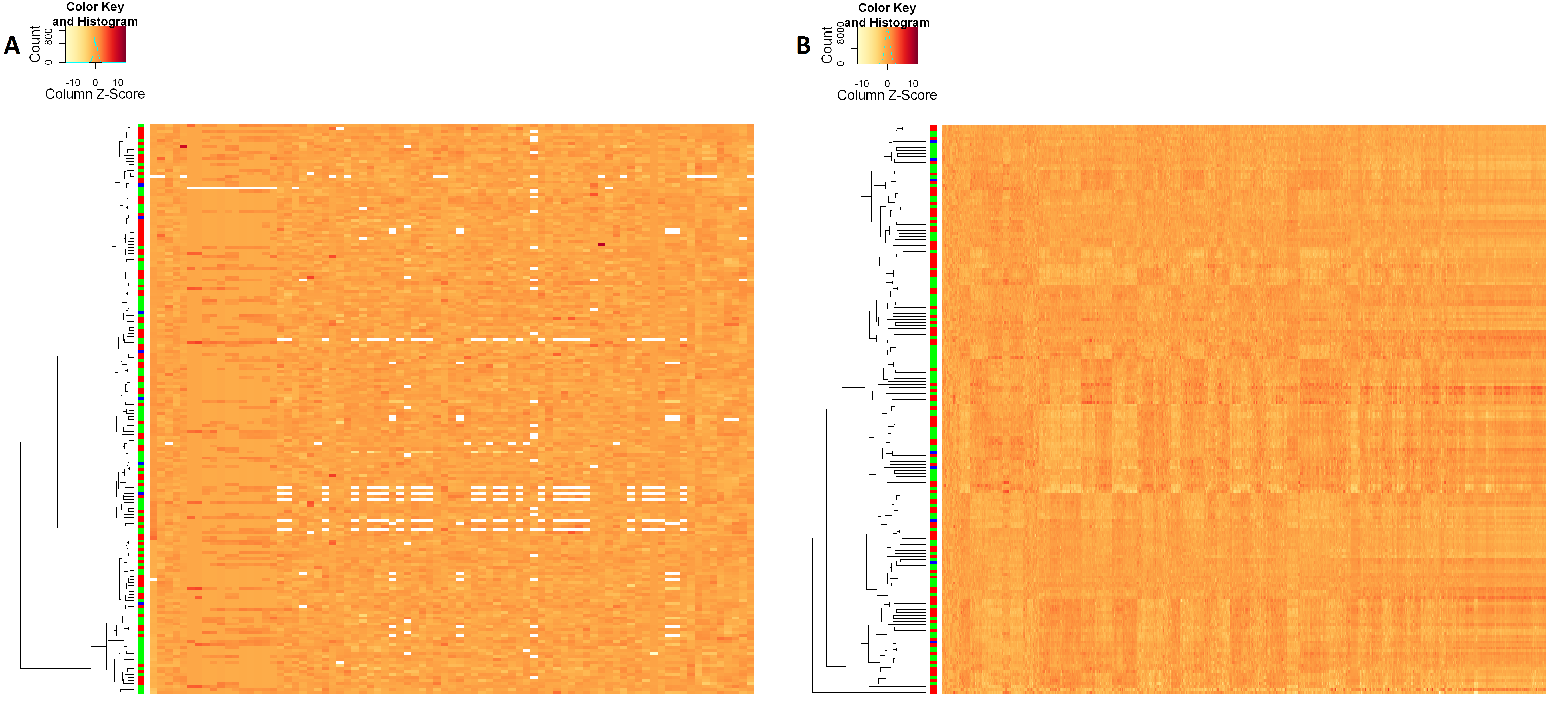

Supplement: S1 Fig — (A) Heatmap showing the 81 clinical biomarkers (X axis) for all subjects (Y axis). (B) Heatmap showing the gene expression (X axis) for 901 genes used in downstream analyses for all 192 subjects (Y axis). For both panels, values are scaled by column using Z scores. Asthma status based on doctor diagnosis is indicated on the left hand side of the heatmap (red = no asthma, green = asthma, blue = unknown). The total number of asthmatics is 96 and the total number of non-asthmatics is 88. Eight individuals did not have a reported asthma status from the doctor diagnosis. (PNG) [file pone.0117445.s001.png]

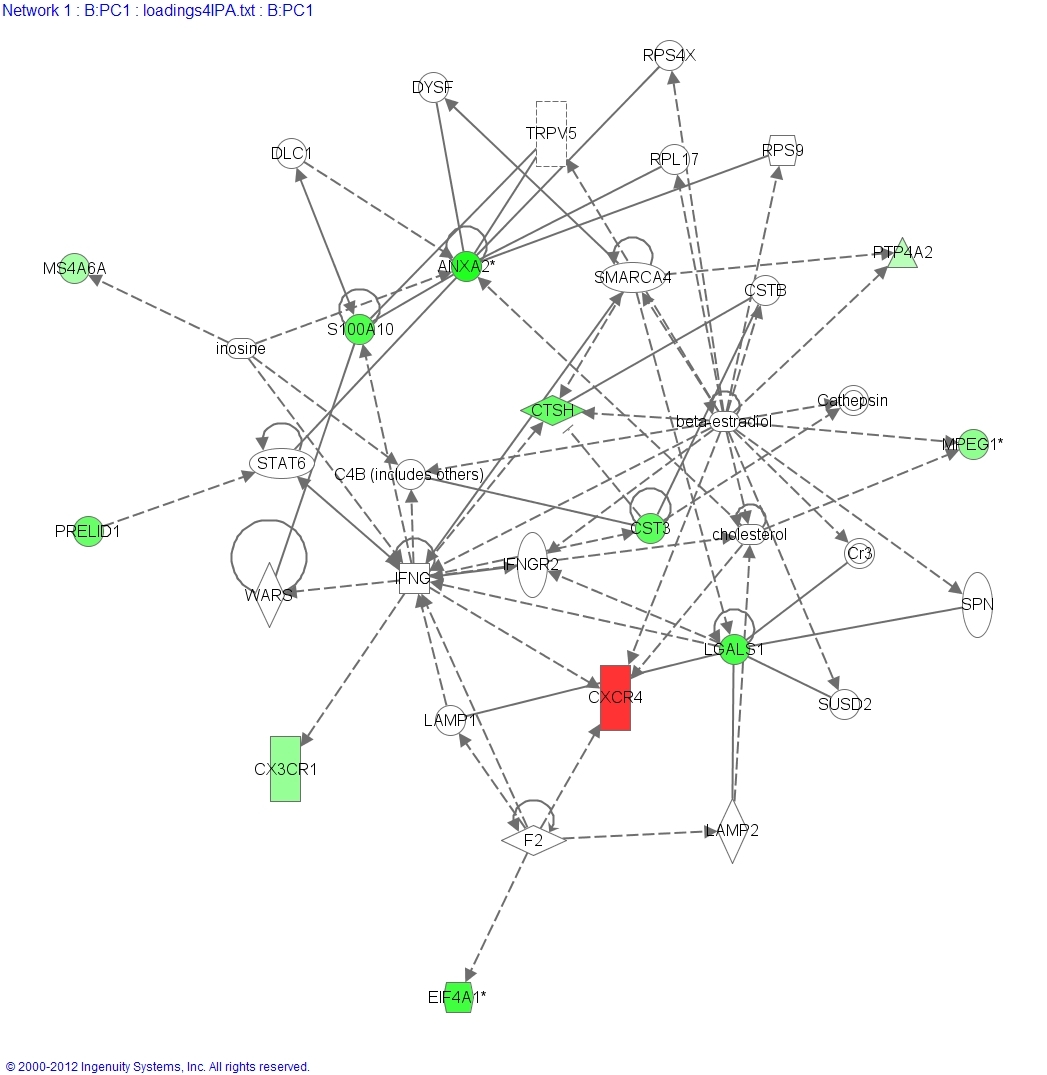

Supplement: S2 Fig — The networks were generated through the use of Ingenuity Pathways Analysis (Ingenuity Systems, www.ingenuity.com). The loading value for each gene was imported in place of an expression value, so green nodes (negative loadings) indicate a gene associated with the left branch (Down) of the metagene whereas red nodes (positive loadings) indicate genes associated with the right branch (Up). (JPG) [file pone.0117445.s002.jpg]

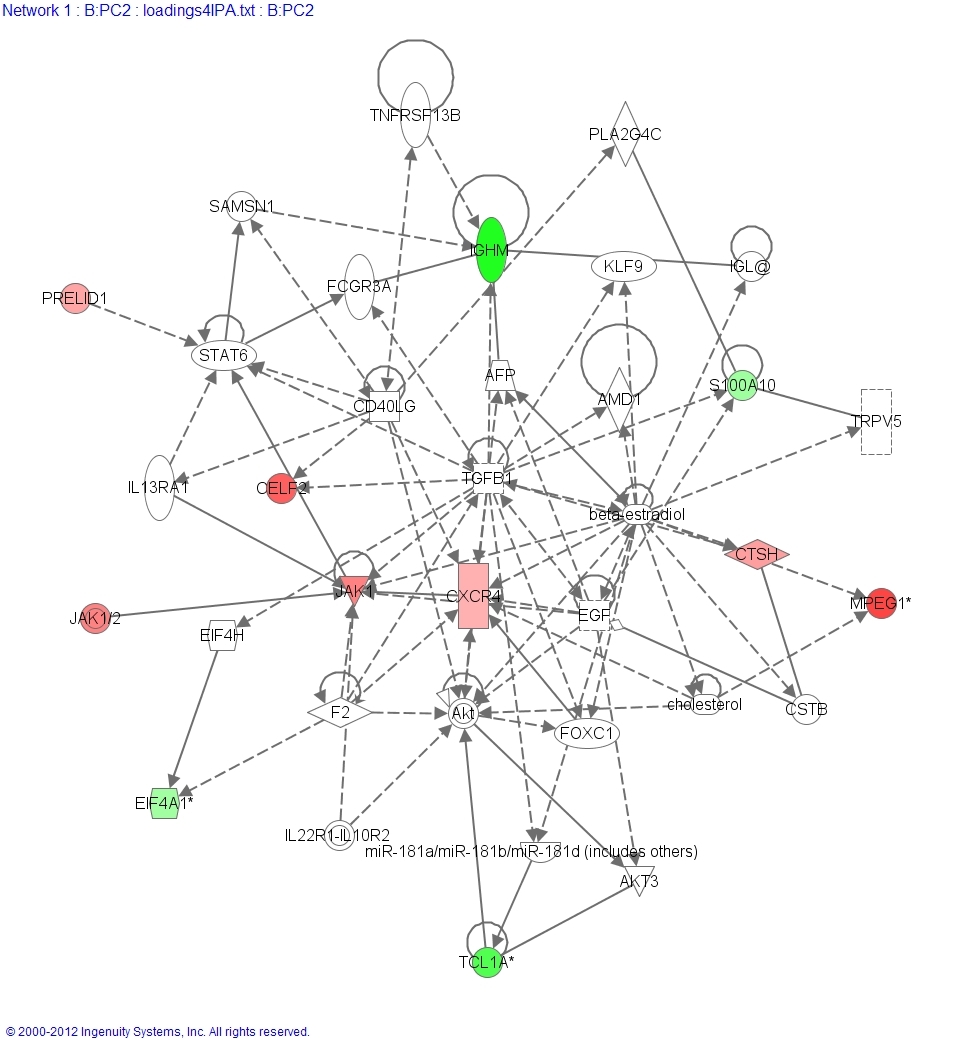

Supplement: S3 Fig — The networks were generated through the use of Ingenuity Pathways Analysis (Ingenuity Systems, www.ingenuity.com). The loading value for each gene was imported in place of an expression value, so green nodes (negative loadings) indicate a gene associated with the left branch (Down) of the metagene whereas red nodes (positive loadings) indicate genes associated with the right branch (Up). (JPG) [file pone.0117445.s003.jpg]

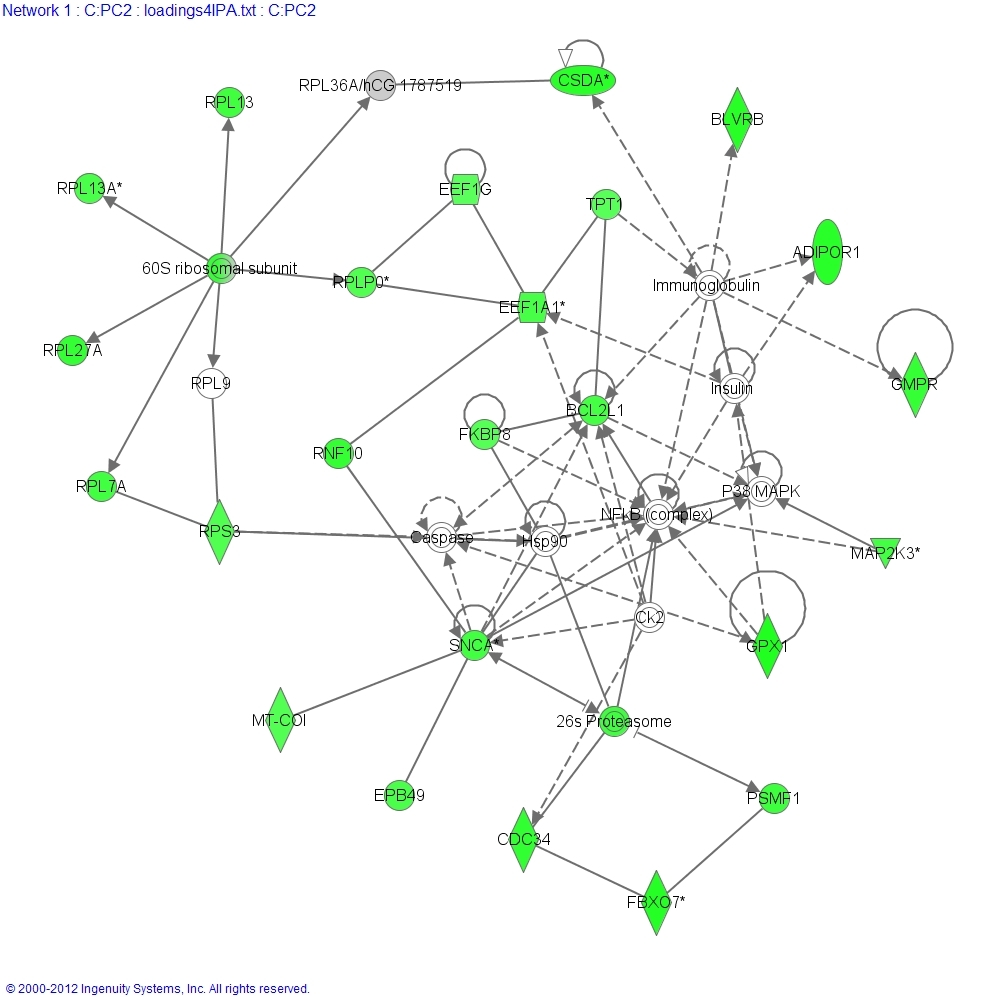

Supplement: S4 Fig — The networks were generated through the use of Ingenuity Pathways Analysis (Ingenuity Systems, www.ingenuity.com). The loading value for each gene was imported in place of an expression value, so green nodes (negative loadings) indicate a gene associated with the left branch (Down) of the metagene whereas red nodes (positive loadings) indicate genes associated with the right branch (Up). (JPG) [file pone.0117445.s004.jpg]

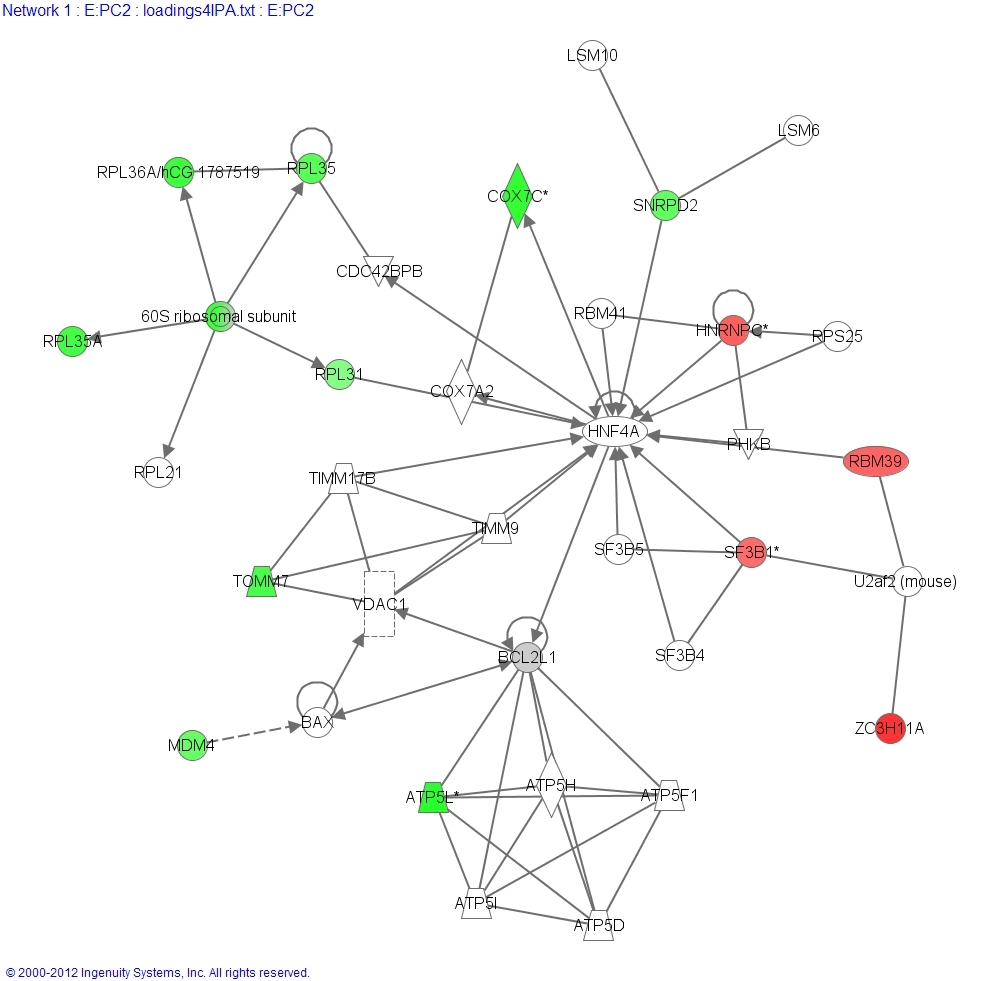

Supplement: S5 Fig — The networks were generated through the use of Ingenuity Pathways Analysis (Ingenuity Systems, www.ingenuity.com). The loading value for each gene was imported in place of an expression value, so green nodes (negative loadings) indicate a gene associated with the left branch (Down) of the metagene whereas red nodes (positive loadings) indicate genes associated with the right branch (Up). See S6 Fig. for the second highest scoring IPA network for E:PC2 since its score was still reasonably high relative to the top scoring network (S3 Table). (JPG) [file pone.0117445.s005.jpg]

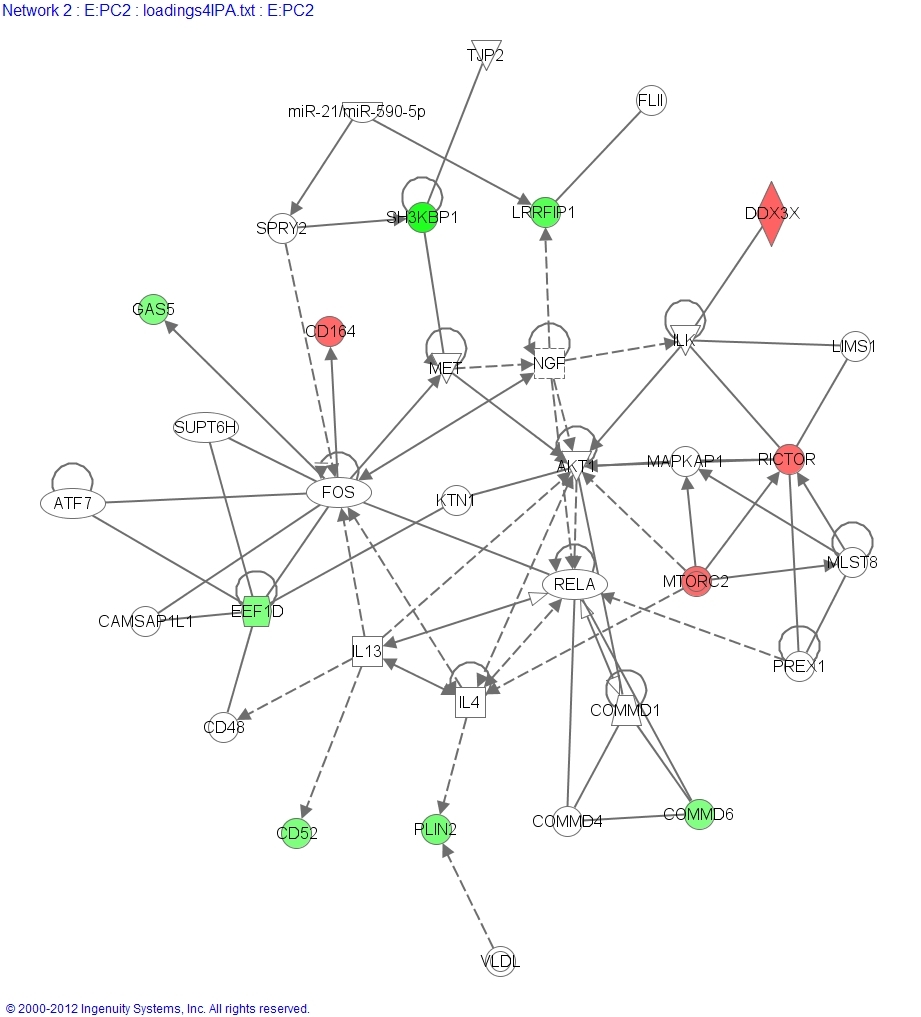

Supplement: S6 Fig — The networks were generated through the use of Ingenuity Pathways Analysis (Ingenuity Systems, www.ingenuity.com). The loading value for each gene was imported in place of an expression value, so green nodes (negative loadings) indicate a gene associated with the left branch (Down) of the metagene whereas red nodes (positive loadings) indicate genes associated with the right branch (Up). Second highest scoring IPA network for E:PC2 since its score was still reasonably high relative to the top scoring network (S3 Table). (JPG) [file pone.0117445.s006.jpg]

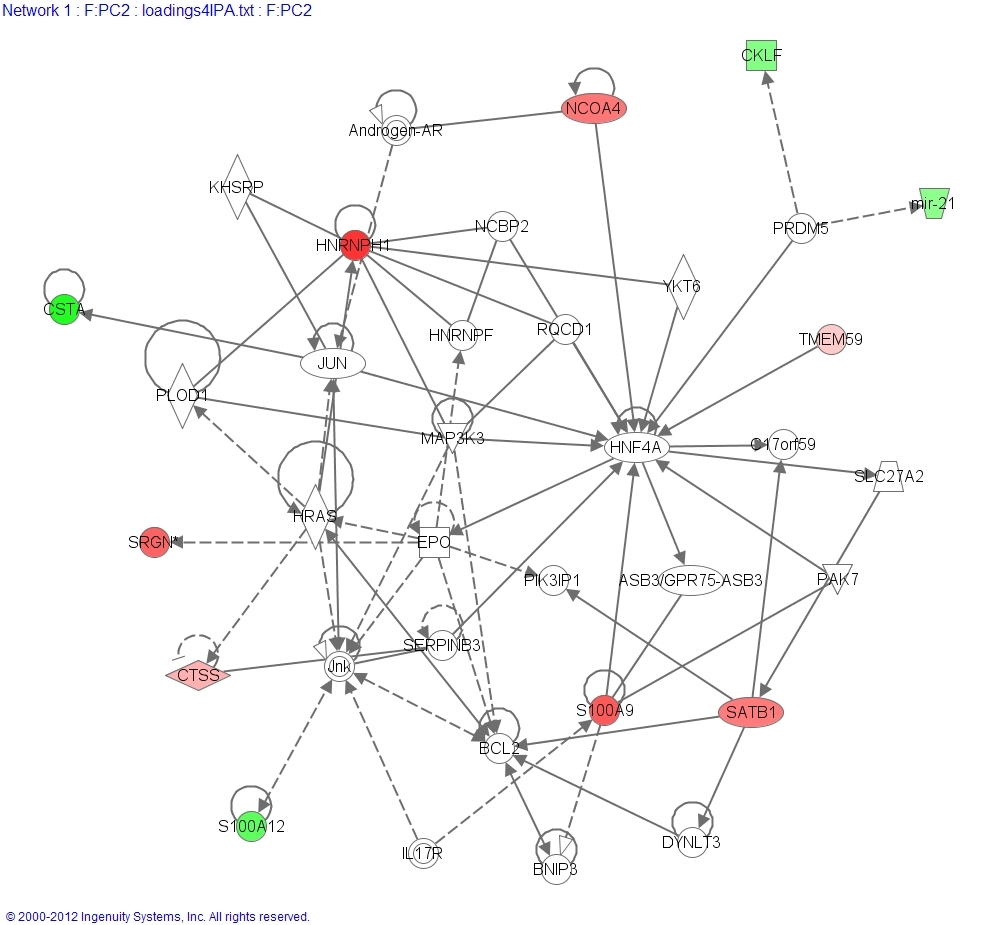

Supplement: S7 Fig — The networks were generated through the use of Ingenuity Pathways Analysis (Ingenuity Systems, www.ingenuity.com). The loading value for each gene was imported in place of an expression value, so green nodes (negative loadings) indicate a gene associated with the left branch (Down) of the metagene whereas red nodes (positive loadings) indicate genes associated with the right branch (Up). (JPG) [file pone.0117445.s007.jpg]

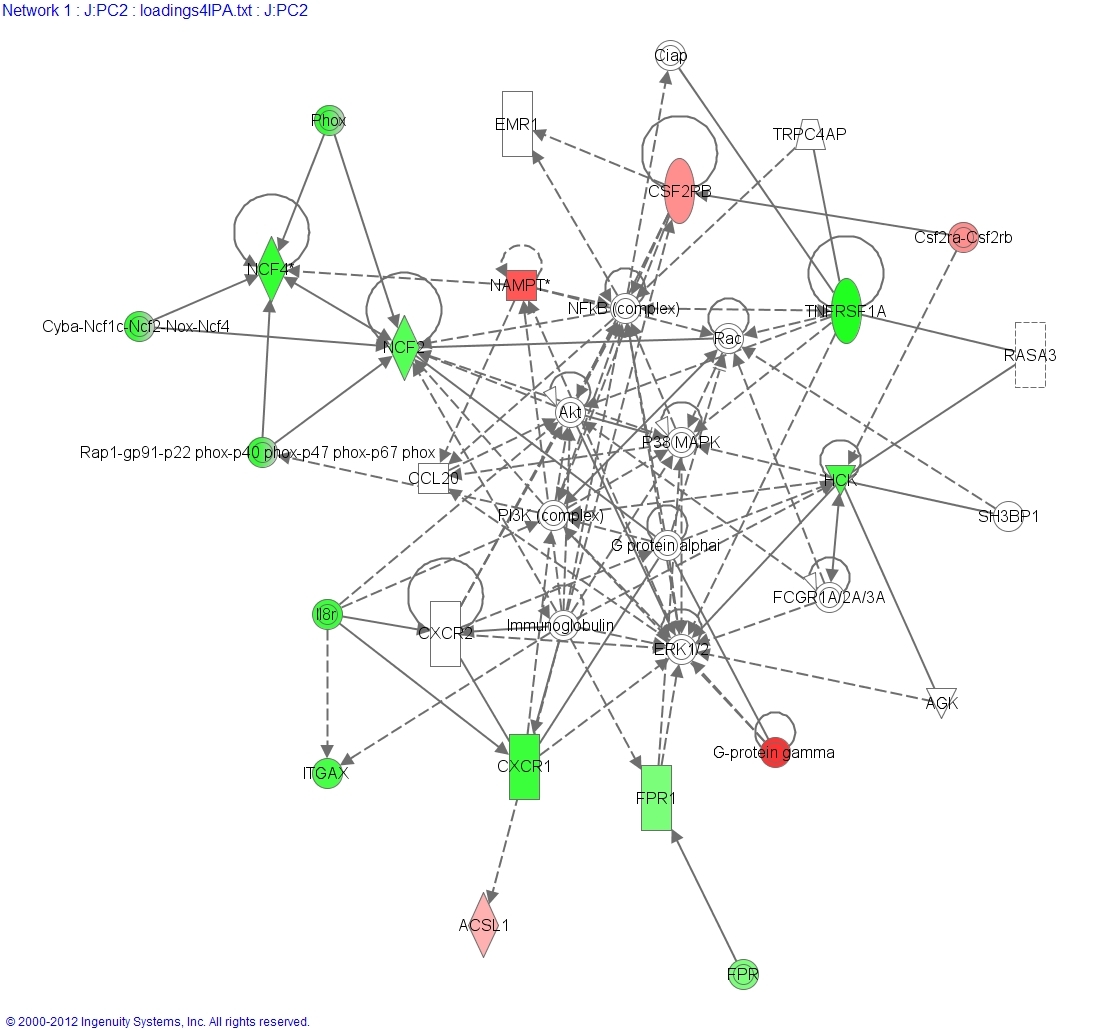

Supplement: S8 Fig — The networks were generated through the use of Ingenuity Pathways Analysis (Ingenuity Systems, www.ingenuity.com). The loading value for each gene was imported in place of an expression value, so green nodes (negative loadings) indicate a gene associated with the left branch (Down) of the metagene whereas red nodes (positive loadings) indicate genes associated with the right branch (Up). See S9 Fig. for the second highest scoring IPA network for J:PC2 since its score was still reasonably high relative to the top scoring network (S3 Table). (JPG) [file pone.0117445.s008.jpg]

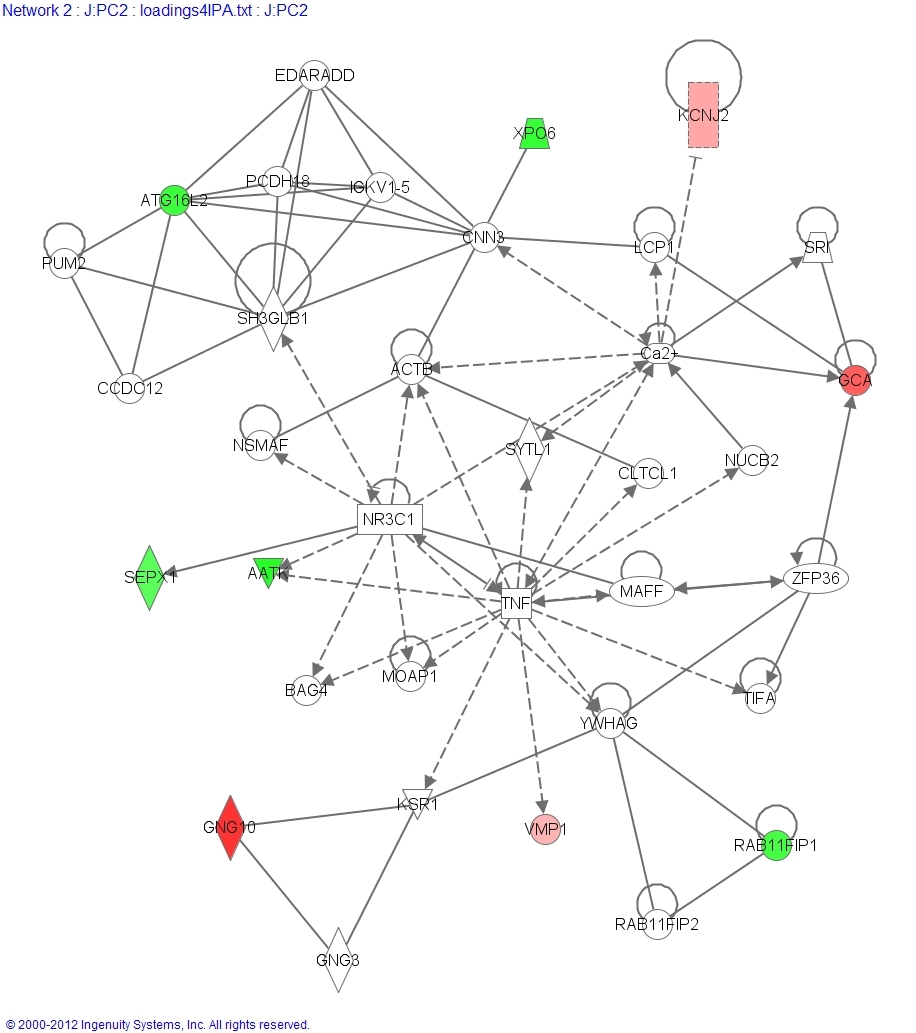

Supplement: S9 Fig — The networks were generated through the use of Ingenuity Pathways Analysis (Ingenuity Systems, www.ingenuity.com). The loading value for each gene was imported in place of an expression value, so green nodes (negative loadings) indicate a gene associated with the left branch (Down) of the metagene whereas red nodes (positive loadings) indicate genes associated with the right branch (Up). Second highest scoring IPA network for J:PC2 since its score was still reasonably high relative to the top scoring network (S3 Table). (JPG) [file pone.0117445.s009.jpg]
