# Supplementary material for: Data-Driven Asthma Endotypes Defined from Blood Biomarker and Gene Expression Data
Source: PLoS One. 2015 Feb 2;10(2):e0117445. doi: 10.1371/journal.pone.0117445 (PMC4314082; doi:10.1371/journal.pone.0117445)
Supplement: S1 Table — Results from a forward-reverse multi-step regression of each metagene from the asthma decision tree on the white blood cell counts and percentage of each individual cell type. Overall contribution of relative changes in cell type was based on the Adjusted R2 coefficient of determination. The sign of the parameter estimate was used to determine the branch of the tree associated with the cell type. (DOCX) [file pone.0117445.s010.docx]

**Table S1.** Contribution of cell type to metagene summarized expression. Results from a forward-reverse multi-step regression of each metagene from the asthma decision tree on the white blood cell counts and percentage of each individual cell type. Overall contribution of relative changes in cell type was based on the Adjusted R^2^ coefficient of determination. The sign of the parameter estimate was used to determine the branch of the tree associated with the cell type.

| **Metagene*** | **Covariate** | **Parameter Estimate** | **p-value** | **Adjusted R^2^** | **Condition Index^ǂ^** |
| --- | --- | --- | --- | --- | --- |
| K-PC1 | Intercept | -0.06 | 0.8288 | 0.6167 | 1.00 |
| (Leaves 1-8) | Percent Blood Lymphocytes | 0.04 | <.0001 |  | 3.27 |
|  | Percent Blood Eosinophils | -0.43 | <.0001 |  | 9.67 |
|  |  |  |  |  |  |
| B-PC2 | Intercept | 3.18 | <.0001 | 0.3656 | 1.00 |
| (Leaves 1-3) | Percent Blood Lymphocytes | -0.12 | <.0001 |  | 6.65 |
|  | Percent Blood Monocytes | 0.19 | 0.0001 |  | 10.64 |
|  |  |  |  |  |  |
|  |  |  |  |  |  |
| B-PC1 | Intercept | 3.24 | <.0001 | 0.3508 | 1.00 |
| (Leaves 4-8) | Percent Blood Lymphocytes | 0.04 | 0.0066 |  | 6.65 |
|  | Percent Blood Monocytes | -0.67 | <.0001 |  | 10.64 |
|  |  |  |  |  |  |
| C-PC2 | (no model)^ |  |  |  |  |
| (Leaves 2-3) |  |  |  |  |  |
|  |  |  |  |  |  |
| J-PC2 | (no model)^ |  |  |  |  |
| (Leaves 4-6) |  |  |  |  |  |
|  |  |  |  |  |  |
| F-PC2 | Intercept | -8.34 | 0.0029 | 0.0484 | 1.00 |
| (Leaves 7-8) | Percent Blood Lymphocytes | 0.11 | 0.0014 |  | 5.33 |
|  | Percent Blood Neutrophils | 0.08 | 0.0068 |  | 55.62 |
|  |  |  |  |  |  |
| E-PC2 | Intercept | -4.27 | 0.0003 | 0.0638 | 1.00 |
| (Leaves 4-5) | Percent Blood Neutrophils | 0.09 | 0.0002 |  | 9.07 |

* Notation for metagenes is Cluster Letter followed by Principal Component (i.e. Cluster K, Principal Component 1 = K-PC1)

**^ǂ^** Collinearity diagnostic where 10 may indicate weak dependencies and increasing severity with higher indexes

^ No variable met the 0.2000 significance level for entry into the model.
