# Supplementary material for: Data-Driven Asthma Endotypes Defined from Blood Biomarker and Gene Expression Data
Source: PLoS One. 2015 Feb 2;10(2):e0117445. doi: 10.1371/journal.pone.0117445 (PMC4314082; doi:10.1371/journal.pone.0117445)
Supplement: S2 Table — B-PC1 and F-PC2 had only one significant network each. For all other metagenes, the highest scoring network that was not considered is included and the score shown. Full page views of all networks considered in the interpretation phase are shown in S2-S9 Figs. (DOCX) [file pone.0117445.s011.docx]

**Table S2.** Top scoring Ingenuity Pathways Analysis networks with associated annotations. B-PC1 and F-PC2 had only one significant network each. For all other metagenes, the highest scoring network that was not considered is included and the score shown. Full page views of all networks considered in the interpretation phase are shown in Figures S2-S9.

| Metagene | Network | | | Associated Network Functions |
| --- | --- | --- | --- | --- |
|  | Rank | Score | Figure |  |
|  |  |  |  |  |
| B-PC1 | 1 | 35 | S2 | Cellular Movement, Hematological System Development and Function, Immune Cell Trafficking |
|  |  |  |  |  |
| B-PC2 | 1 | 27 | S3 | Cell-to-Cell Signaling and Interaction, Cell Death, Cellular Growth and Proliferation |
|  | 2 | 6 |  | Not considered |
|  |  |  |  |  |
| C-PC2 | 1 | 66 | S4 | Protein Synthesis, Cell Death, Gene Expression |
|  | 2 | 24 |  | Not considered |
|  |  |  |  |  |
| E-PC2 | 1 | 33 | S5 | Protein Synthesis, RNA Post-Transcriptional Modification, DNA Replication, Recombination, and Repair |
|  | 2 | 24 | S6 | Gene Expression, Cellular Development, Cellular Growth and Proliferation |
|  | 3 | 3 |  | Not considered |
|  |  |  |  |  |
| F-PC2 | 1 | 33 | S7 | Cardiac Necrosis/Cell Death, Cell Death, Gene Expression |
|  |  |  |  |  |
| J-PC2 | 1 | 24 | S8 | Free Radical Scavenging, Cell-to-Cell Signaling and Interaction, Hematological System Development and Function |
|  | 2 | 21 | S9 | Cellular Assembly and Organization, Gene Expression, Cardiovascular Disease |
|  | 3 | 3 |  | Not considered |
