# Supplementary material for: Data-Driven Asthma Endotypes Defined from Blood Biomarker and Gene Expression Data
Source: PLoS One. 2015 Feb 2;10(2):e0117445. doi: 10.1371/journal.pone.0117445 (PMC4314082; doi:10.1371/journal.pone.0117445)
Supplement: S3 Table — Geometric means and CIs were calculated using SAS-callable SUDAAN [57]; other statistics were calculated using SAS [58]. (DOCX) [file pone.0117445.s012.docx]

**Table S3**. Inflammation/allergy-related characteristics of subjects by leaf (*potential asthma endotype)

| **Leaf** | **1*** | **2*** | **3** | **4** | **5*** | **6** | **7** | **8*** |
| --- | --- | --- | --- | --- | --- | --- | --- | --- |
| Sample Size (n) | 30 | 14 | 15 | 14 | 15 | 29 | 14 | 15 |
| Asthma | 90.0 | 71.4 | 26.7 | 21.4 | 60.0 | 6.9 | 42.9 | 73.3 |
| Median Age (Yrs) | 12.0 | 11.2 | 12.0 | 11.6 | 11.7 | 11.3 | 11.9 | 11.3 |
| Male | 63.3 | 71.4 | 66.7 | 71.4 | 46.7 | 41.4 | 71.4 | 46.7 |
| African-American | 83.3 | 71.4 | 53.3 | 64.3 | 80.0 | 86.2 | 85.7 | 80.0 |
| Atopic | 96.6 | 71.4 | 100.0 | 35.7 | 53.3 | 41.4 | 71.4 | 33.3 |
| Eosinophilic | 43.3 | 28.6 | 53.3 | 7.1 | 0.0 | 0.0 | 0.0 | 0.0 |
| **Geometric Mean** | **(95% CI)** |  |  |  |  |  |  |  |
| Total Serum IgE (kU/L) | 348.9  (204.6, 493.2) | 117.7 (43.4, 192.0) | 218.8  (117.7, 319.9) | 42.0  (5.3, 78.8) | 67.4  (15.3, 119.6) | 49.0  (20.2, 77.8) | 131.2  (17.5, 244.9) | 36.1  (3.4, 68.8) |
| Phadiatop  (kUA/L) | 23.9  (10.6, 37.2) | 3.5  (0.0, 7.9) | 17.1  (5.0, 29.3) | 1.1  (0.0, 2.6) | 1.6  (0.0, 3.5) | 0.9  (0.2, 1.7) | 2.7  (0.0, 5.7) | 0.9  (0.0, 1.9) |
| Food Screen Fx5e (kUA/L) | 1.6  (0.5, 2.8) | 0.3  (0.2, 0.5) | 0.4  (0.2, 0.5) | 0.3  (0.0, 0.6) | 0.3  (0.0, 0.6) | 0.4  (0.2, 0.5) | 0.4  (0.1, 0.7) | 0.2  (0.1, 0.3) |
| Blood Eosinophils (K/uL) | 0.4  (0.3, 0.4) | 0.3  (0.2, 0.4) | 0.4  (0.3, 0.5) | 0.2  (0.1, 0.2) | 0.1  (0.1, 0.2) | 0.1  (0.1, 0.2) | 0.1  (0.1, 0.2) | 0.1  (0.1, 0.1) |
| Blood Neutrophils (K/uL) | 2.7  (2.2, 3.1) | 3.3  (2.4, 4.3) | 4.0  (3.2, 4.9) | 2.4  (2.1, 2.6) | 3.7  (3.0, 4.4) | 3.0  (2.4, 3.6) | 3.2  (2.2, 4.1) | 3.4  (3.1, 3.8) |
| Blood Lymphocytes (K/uL) | 2.7  (2.4, 2.9) | 2.2  (2.0, 2.5) | 2.7  (2.5, 3.0) | 2.5  (2.4, 2.7) | 2.4  (2.1, 2.8) | 2.7  (2.4, 2.9) | 2.5  (2.0, 2.9) | 2.6  (2.3, 2.9) |
| Blood Monocytes (K/uL) | 0.4  (0.4, 0.5) | 0.5  (0.4, 0.6) | 0.5  (0.4, 0.6) | 0.4  (0.4, 0.5) | 0.5  (0.4, 0.6) | 0.5  (0.5, 0.6) | 0.3  (0.3, 0.4) | 0.4  (0.3, 0.4) |
| Total White Blood Cells (K/uL) | 6.3  (5.7, 7.0) | 6.8  (5.6, 7.9) | 7.8  (6.7, 8.9) | 5.5  (5.2, 5.9) | 6.9  (5.9, 7.9) | 6.5  (5.7, 7.3) | 6.5  (5.5, 7.5) | 6.6  (6.1, 7.1) |
| Exhaled nitric oxide (ppb) | 27.2  (18.3, 36.1) | 21.6  (11.6, 31.5) | 21.7  (9.7, 33.7) | 10.1  (6.9, 13.3) | 13.3  (8.7, 17.9) | 10.5  (6.8, 14.1) | 14.1  (7.4, 20.7) | 8.4  (5.3, 11.5) |

Note: Selected demographics (top) are reported as percentages unless noted. The geometric means and 95% confidence intervals (CIs) for inflammation and allergy-related biomarkers (bottom) for each leaf in the decision tree (Figure 1B). Asthma status of the subjects was determined by doctor diagnosis; *indicates leaf where the majority of the subjects have asthma. Phadiatop is a single analysis that measures the relative level of IgE antibody specific for a panel of 10 common aeroallergens. Subjects were defined as atopic when phadiatop >= 0.35 kUA/L. Eosinophilia was defined as blood eosinophils > 0.4 K/uL. Total Serum IgE (kU/L) <2.0 was changed to 1. Phadiatop (kUA/L) and Fx5e Food Screen (kUA/L) values <0.35 were changed to 0.175 and >100 were changed to 101. The 95% CIs were left truncated at 0. Geometric means and CIs were calculated using SAS-callable SUDAAN [[57](#_ENREF_1)]; other statistics were calculated using SAS [[58](#_ENREF_2)].
