# Supplementary material for: Data-Driven Asthma Endotypes Defined from Blood Biomarker and Gene Expression Data
Source: PLoS One. 2015 Feb 2;10(2):e0117445. doi: 10.1371/journal.pone.0117445 (PMC4314082; doi:10.1371/journal.pone.0117445)
Supplement: S4 Table — (DOCX) [file pone.0117445.s013.docx]

**Table S4.** Metabolic syndrome-related clinical markers along with lung function and medication use by leaf (*potential asthma endotype)

| **Leaf** | **1*** | **2*** | **3** | **4** | **5*** | **6** | **7** | **8*** |
| --- | --- | --- | --- | --- | --- | --- | --- | --- |
| Sample Size (n) | 30 | 14 | 15 | 14 | 15 | 29 | 14 | 15 |
| Asthma (n) | 27 | 10 | 4 | 3 | 9 | 2 | 6 | 11 |
| No asthma (n) | 3 | 4 | 11 | 11 | 6 | 27 | 8 | 4 |
| **Geometric Mean (95% CI)** | | | | | | | | |
| BMI [weight(kg)/  height(m)2 ] | 21.8  (19.9, 23.7) | 21.9  (18.5, 25.3) | 21.4  (19.3, 23.6) | 18.3  (16.3, 20.4) | 21.3  (18.8, 23.7) | 23.6  (21.4, 25.8) | 19.4  (16.6, 22.1) | 21.7  (19.3, 24.2) |
| Serum Triglycerides (mg/dL) | 73.4  (62.3, 84.6) | 82.4  (67.5, 97.3) | 85.9  (66.9, 105.0) | 62.9  (53.7, 72.2) | 66.7  (56.8, 76.5) | 79.3  (66.4, 92.2) | 68.3  (57.1, 79.5) | 76.3  (58.6, 94.0) |
| Serum VLDL (mg/dL) | 14.7  (12.4, 16.9) | 16.5  (13.6, 19.4) | 17.2  (13.5, 21.0) | 12.5  (10.7, 14.3) | 13.2  (11.1, 15.3) | 15.9  (13.3, 18.5) | 13.5  (11.3, 15.8) | 15.2  (11.7, 18.7) |
| Plasma Leptin (ng/ml) | 1.7  (0.8, 2.6) | 1.9  (0.8, 3.0) | 2.0  (0.5, 3.5) | 2.1  (0.8, 3.5) | 1.8  (0.7, 2.9) | 3.5  (2.2, 4.9) | 1.4  (0.4, 2.4) | 2.4 (0.4, 4.3) |
| FEV1.0 / FVC ratio | 79.9  (76.3, 83.6) | 79.7  (74.4, 84.9) | 86.7  (84.5, 88.8) | 84.3  (82.3, 86.2) | 81.2  (75.7, 86.8) | 86.3  (84.0, 88.7) | 81.8  (78.6, 84.9) | 85.2  (82.7, 87.7) |
| **Asthma Medication Use (% asthmatics)** | | | | | | | | |
| None | 3.7 | 20.0 | 0.0 | 0.0 | 11.1 | 0.0 | 0.0 | 18.2 |
| Occasional | 48.2 | 20.0 | 75.0 | 33.3 | 22.2 | 0.0 | 33.3 | 36.4 |
| Daily | 18.5 | 30.0 | 25.0 | 66.7 | 44.4 | 100.0 | 50.0 | 27.3 |
| 2 Meds Daily | 29.6 | 30.0 | 0.0 | 0.0 | 22.2 | 0.0 | 16.7 | 18.2 |

Note: Geometric means and 95% confidence intervals (CIs) per leaf in the decision tree for selected metabolic syndrome-related biomarkers found correlated with informative metagenes along with lung function and medication use. The 95% CIs were left truncated at 0. Geometric means and CIs were calculated using SAS-callable SUDAAN [[57](#_ENREF_1)]; other statistics were calculated using SAS [[58](#_ENREF_2)]. Medication use was calculated as the percentage of the total asthmatics in the group falling into each category.
