# Supplementary material for: Data-Driven Asthma Endotypes Defined from Blood Biomarker and Gene Expression Data
Source: PLoS One. 2015 Feb 2;10(2):e0117445. doi: 10.1371/journal.pone.0117445 (PMC4314082; doi:10.1371/journal.pone.0117445)
Supplement: S6 Table — (DOCX) [file pone.0117445.s015.docx]

**Table S6.** Biomarker listing with rows and groups as in Figure 1A dendogram

| Group | Row | Description | Units | | Annotation | |
| --- | --- | --- | --- | --- | --- | --- |
| 1 | 1 | Serum Total Globulin | | g/dL | |  |
|  | 2 | Albumin/Globulin Ratio | |  | |  |
|  | 3 | Serum Uric Acid | | mg/dL | | Body weight, cholesterol, metabolic indicators |
|  | 4 | Total Cholesterol to High density Lipoprotein Ratio | |  | | Body weight, cholesterol, metabolic indicators |
|  | 5 | Low density Lipoprotein | | mg/dL | | Body weight, cholesterol, metabolic indicators |
|  | 6 | Total Cholesterol | | mg/dL | | Body weight, cholesterol, metabolic indicators |
|  | 7 | Serum Glucose | | mg/dL | | Body weight, cholesterol, metabolic indicators |
|  | 8 | Serum Phospholipids Concentration | | Mg/dL | | Body weight, cholesterol, metabolic indicators |
|  | 9 | Serum Unbound Iron-Binding Capacity | | ug/dL | | Body weight, cholesterol, metabolic indicators |
|  | 10 | Plasma Leptin | | ng/mL | | Body weight, cholesterol, metabolic indicators |
|  | 11 | Blood Pressure Pulse | | beats/min | | Body weight, cholesterol, metabolic indicators |
|  | 12 | Subject Weight | | kg | | Body weight, cholesterol, metabolic indicators |
|  | 13 | Subject Body Mass Index Weight/height | | kg/m^2^ | | Body weight, cholesterol, metabolic indicators |
|  | 14 | Very Low Density Lipoprotein | |  | | Body weight, cholesterol, metabolic indicators |
|  | 15 | Serum Triglycerides | | mg/dL | | Body weight, cholesterol, metabolic indicators |
| 2 | 1 | Serum Derm Farin Dustmite | | kUA/L | | Allergens |
|  | 2 | Serum Derm Pter Dustmite | | kUA/L | | Allergens |
|  | 3 | Phadiatop (10 common allergy screen)^#^ | | kUA/L | | Allergens |
|  | 4 | Fractional Exhaled Nitric Oxide | |  | |  |
|  | 5 | Serum Chloride | | mmol/L | |  |
|  | 6 | BP Oxygen Saturation (Dissolved Oxygen) | | % | |  |
|  | 7 | Blood Hemoglobin | | g/DdL | |  |
|  | 8 | High density Lipoprotein | | mg/dL | |  |
|  | 9 | Urine Creatinine | | mg/dL | |  |
|  | 10 | Platelet Count | | K/uL | |  |
|  | 11 | Serum Creatinine | | mg/dL | |  |
|  | 12 | Serum Glycated Hemoglobin | | % | |  |
|  | 13 | Serum Aspartate Aminotranferase (AST) Serum Glutamic-Oxaloacetic Transaminase (SGOT) | | IU/L | |  |
|  | 15 | Serum Blood Urea Nitrogen | | mg/dL | |  |
|  | 16 | Serum Total Protein | | g/dL | |  |
|  | 17 | Mean of first Two Diastolic Blood Pressure Measurements | | mmHg | |  |
|  | 18 | Serum Ferritin | | ng/ml | |  |
|  | 19 | Serum Gamma-Glutamyl transpeptidase (GGT) | | IU/L | |  |
|  | 20 | Serum Lactate Dehydrogenase | | IU/L | |  |
|  | 21 | Serum Total Bilirubin | | mg/dL | |  |
|  | 22 | Forced Expiratory Volume 1 ratio to Forced Vital Capacity | | % | | Lung Function |
|  | 23 | Forced Expiratory Volume 5 ratio to Forced Vital Capacity | | % | | Lung Function |
|  | 24 | Peak Expiratory Flow | | L/min | | Lung Function |
|  | 25 | Forced Expiratory Flow Between 25% and 75% of Forced Expiratory Flow | |  | | Lung Function |
|  | 26 | Sodium | | mmol/L | |  |
|  | 27 | Serum Osmolality | | mOsmol/kg | |  |
|  | 28 | Serum Calcium | | mg/dL | |  |
|  | 29 | Albumin | | g/dL | |  |
|  | 30 | Serum German Cockroach | | kUA/L | |  |
|  | 31 | Alkaline phosphatase | | IU/L | |  |
|  | 32 | Serum Total Antioxident Status | | mmol/L | |  |
|  | 33 | Serum Glutamic Pyruvic Transaminase | | IU/L | |  |
|  | 34 | Potassium | | mmol/L | |  |
|  | 35 | Serum Arachidonic Acid | | ug/ml | |  |
|  | 36 | Serum Phosphorus | | mg/dL | |  |
|  | 37 | Blood Hematocrit | | % | |  |
|  | 38 | Subject Height | | cm | |  |
|  | 39 | Subject Age | | Years | |  |
|  | 40 | Serum Iron | | ug/dl | |  |
|  | 41 | C-reactive Protiein | | mg/mL | |  |
|  | 42 | Total Serum IgE | | kU/L | | Allergens |
|  | 43 | FoodScreen (5 common food allergens) | | kUA/L | | Allergens |
|  | 44 | Serum Cat Dander Epithel | | kUA/L | | Allergens |
|  | 45 | Serum Dog Dander | | kUA/L | | Allergens |
|  | 46 | Serum Alternaria Alternata | | kUA/L | | Allergens |
|  | 47 | Serum Aspergillus Fumigatus | | kUA/L | | Allergens |
|  | 48 | Mean of first two Systolic Blood Pressure Measurements | | mmHg | |  |
|  | 49 | Red Blood Cell Distribution Width | | % | |  |
|  | 50 | Serum Rat Urine Protein | | kUA/L | | Allergens |
|  | 51 | Serum Mouse Urine Protein | | kUA/L | | Allergens |
|  | 52 | Serum Cladosporium Herbarum | | kUA/L | | Allergens |
|  | 53 | Serum Penicillium Notatum | | kUA/L | | Allergens |
|  | 54 | Basophil percent of sum White Blood Cells | | % | |  |
|  | 55 | Plasma Average of Reactive Oxygen Species Measurments minus Control | | RLU* | |  |
|  | 56 | Interleukin-4 | | pg/ml | |  |
|  | 57 | Serum Fibrinogen | | mg/dL | |  |
| 3 | 1 | Mean Corpuscular Volume | | fL** | |  |
|  | 2 | Mean Corpuscular Hemoglobin | | pg | |  |
|  | 3 | Red Blood Cell Count | | M/uL | | Blood cell count |
|  | 4 | Mean Corpuscular hemoglobin concentration | | g/dL | | Blood cell count |
|  | 5 | Eosinophil percent of sum White Blood Cells | | % | | Blood cell count |
|  | 6 | Monocyte percent of sum White Blood Cells | | % | | Blood cell count |
| 4 | 1 | White Blood Cell Count | | K/uL | | Blood cell count |
|  | 2 | Neutrophils percent of sum White Blood Cells | | % | | Blood cell count |
|  | 3 | Lymphocyte percent of sum White Blood Cells | | % | | Blood cell count |

* relative luminescence unit, ** femtoliters. ^#^Phadiatop is a single analysis that measures the relative level of IgE antibody specific for a panel of 10 common aeroallergens. The Group column is color-coded to match the dendrogram on the Y-axis of Figure 1A; row designates the order of its biomarkers. Annotation column shows the precise biomarkers referenced by the annotations provided on Figure 1A.
